# Role of miRNAs in sucrose stress response, reactive oxygen species, and anthocyanin biosynthesis in *Arabidopsis thaliana*

**DOI:** 10.3389/fpls.2023.1278320

**Published:** 2023-11-03

**Authors:** Md. Fakhrul Azad, Pranav Dawar, Nevzat Esim, Christopher D. Rock

**Affiliations:** ^1^Department of Biological Sciences, Texas Tech University, Lubbock, TX, United States; ^2^Department of Molecular Biology and Genetics, Bіngöl University, Bingöl, Türkiye

**Keywords:** microRNA, sucrose stress, miRNA target, degradome, polyphenolic, secondary metabolism, oxidative stress

## Abstract

In plants, sucrose is the main transported disaccharide that is the primary product of photosynthesis and controls a multitude of aspects of the plant life cycle including structure, growth, development, and stress response. Sucrose is a signaling molecule facilitating various stress adaptations by crosstalk with other hormones, but the molecular mechanisms are not well understood. Accumulation of high sucrose concentrations is a hallmark of many abiotic and biotic stresses, resulting in the accumulation of reactive oxygen species and secondary metabolite anthocyanins that have antioxidant properties. Previous studies have shown that several MYeloBlastosis family/MYB transcription factors are positive and negative regulators of sucrose-induced anthocyanin accumulation and subject to microRNA (miRNA)–mediated post-transcriptional silencing, consistent with the notion that miRNAs may be “nodes” in crosstalk signaling by virtue of their sequence-guided targeting of different homologous family members. In this study, we endeavored to uncover by deep sequencing small RNA and mRNA transcriptomes the effects of exogenous high sucrose stress on miRNA abundances and their validated target transcripts in Arabidopsis. We focused on genotype-by-treatment effects of high sucrose stress in *Production of Anthocyanin Pigment 1-Dominant/pap1-D*, an activation-tagged dominant allele of MYB75 transcription factor, a positive effector of secondary metabolite anthocyanin pathway. In the process, we discovered links to reactive oxygen species signaling through miR158/161/173-targeted *Pentatrico Peptide Repeat* genes and two novel non-canonical targets of high sucrose-induced miR408 and miR398b*(star), relevant to carbon metabolic fluxes: *Flavonoid 3’-Hydroxlase (F3’H)*, an important enzyme in determining the B-ring hydroxylation pattern of flavonoids, and *ORANGE* a post-translational regulator of Phytoene Synthase expression, respectively. Taken together, our results contribute to understanding the molecular mechanisms of carbon flux shifts from primary to secondary metabolites in response to high sugar stress.

## Introduction

Plant sugars, specifically sucrose, are the mobile photosynthetic “end product” transported from the vegetative source to reproductive and/or storage sink organs. Sucrose plays an important role in modulating general metabolism to balance carbon assimilation with macronutrients nitrogen and inorganic phosphate (P_i_) availability (Lei et al., 2011; Dasgupta et al., 2014), in addition to serving as source of energy from respiration which generates reactive oxygen species (ROS) (Koch, 2004). Carbohydrates are the ultimate source of carbon skeletons for building primary metabolites amino acids, lipids, photosynthetic pigments, nucleic acids, and secondary metabolites like polyphenolics involved in environmental stress adaptations. Sugar signaling regulates fundamental processes such as photosynthesis, nutrition mobilization, and source allocation to reproductive and storage sink tissues via homeostatic feedback loops (Horacio and Martinez-Noel, 2013; Román et al., 2021). Elevated sugar levels cause developmental arrest, which has been used to identify mutants through forward genetics in order to explore the processes controlling sugar signaling (Teng et al., 2005; Solfanelli et al., 2006). If rate-limiting links between carbon assimilation and nutrient availability could be elucidated and functionally uncoupled, crop productivity could benefit by engineered carbon partitioning to minimize pleiotropic (synergistic and antagonistic) effects of nutrient use changes directed to sink organs (Yadav et al., 2015).

Plants are sessile and have evolved the ability to integrate all environmental inputs to networked signaling and metabolic pathways. Sugar has hormone-like signaling capabilities that, in addition to directing plant growth and development, also mediate responses to diverse stimuli. The buildup of soluble sugars is a hallmark of biotic and abiotic stresses (Rosa et al., 2009; Jeandet et al., 2022). On the other hand, sugar starvation responses have been modeled by data-driven systems analysis of starch deficient mutants (Arias et al., 2014), and energy deprivation activates sugar-non-fermenting (Snf)–related protein kinase SnRK1 to promote energy homeostasis and adaptive metabolic reprogramming (Rodrigues et al., 2013). Sugar crosstalk with plant stress hormones jasmonic acid and abscisic acid (ABA) leads to expression of stress-inducible and pathogen response–related genes (Finkelstein et al., 2002; León and Sheen, 2003), but it is still unclear how this complex regulatory network is controlled at post transcriptional levels. MicroRNAs (miRNAs) serving as “nodes” in networks of integrated plant external and internal response signaling pathways are a testable hypothesis to better understand molecular mechanisms of plant signaling.

miRNAs are 21- to 24-nucleotide (nt) small-RNA (sRNA) species generated from non-coding hairpin-structured *MIRNA* loci transcribed by RNA polymerase II (Xie et al., 2005). Nascent *MIRNA* transcripts adopt a thermodynamically stable imperfect stem loop hairpin structure known as primary miRNA (pri-miRNA) (Chen, 2009; Li and Yu, 2021). A DICER-LIKE1 (DCL1) endonuclease cuts the ends of the pri-miRNA at the hairpin base (or loop, rarely) to generate a pre-miRNA which subsequently undergoes DCL1-mediated processive cleavages to generate an asymmetrical duplex structure with two nt 3′ overhangs known as miRNA/miRNA* (*=“star/passenger”) duplex (Treiber et al., 2019; Li and Yu, 2021; Dong et al., 2022). This duplex structure subsequently gets methylated at the 2′ free hydroxyl position of 3′ ends by HUA ENHANCER1 (HEN1), whereby methylation stabilizes the complex from hydrolysis and processive activities of exonucleases or adenylases. The stable duplex is then transported to the cytoplasm by HASTY1 exportin, where the mature strand gets incorporated into an ARGONAUTE (AGO) endoribonuclease subunit effector of the RNA-induced silencing complex (Li and Yu, 2021). AGO proteins have PAZ and PIWI domains where the PAZ domain binds to the 3′ end of the miRNA, whereas the PIWI domain has characteristic RNase H endonuclease activity that facilitates the cleavage (“slicing”) of a target mRNA at 10th nt position from the 5′ end of mature miRNA when hybridized with the guide miRNA via Watson-Crick base sequence complementarity (Llave et al., 2002; Ronemus et al., 2006; Li and Yu, 2021).

miRNAs not only mediate negative regulation of gene expression in plants primarily by AGO-slicing activity toward mRNA targets but also can act via transcriptional silencing at the DNA locus of a protein-coding target gene (Bao et al., 2004; Chellappan et al., 2010) and/or translational inhibition of an mRNA (Brodersen et al., 2008). Some miRNAs (mainly 22-nt length) can target non-coding mRNAs called *Trans-Acting-Small-interfering locus (TAS)* to transitively generate and amplify TAS-derived small-interfering RNAs (tasiRNAs) (Ronemus et al., 2006). *TAS* and related *PHAS* loci generate 21 nt tasi- and phasi-RNAs that can cascade by AGO1 association with rough endomembrane-bound polysomes (Li et al., 2016) to target one or more distinct genetic loci different from their locus of origin. Although their small size might suggest accessory roles in function, from a regulatory perspective, miRNAs and siRNAs are big players in gene regulation ranging from growth and development to biotic and abiotic stress responses (D’Ario et al., 2017; Song et al., 2019; Azad et al., 2023).

We approached the question of miRNA functions in plant carbon metabolism and oxidative stress responses by analyzing miRNA and mRNA dynamics in response to high sucrose treatments in model plant *Arabidopsis thaliana* 6-day-old seedings, which respond by accumulating high amounts of antioxidant anthocyanins (Finkelstein et al., 2002). We characterized relative changes by quantitative assays of RNA-seq and sRNA-seq libraries from Arabidopsis Col-0 (control) and *Production of Anthocyanin Pigment 1-Dominant* (*pap1-D*), an activation-tagged line that constitutively overexpresses Arabidopsis *PAP1* (*Production of Anthocyanin Pigment 1*)/*MYB75* transcription factor (Borevitz et al., 2000). The *pap1-D* genotype served as our subject reference for hypothesized miRNA gene-by-environment interactions because it is targeted by a *MIR828:TAS4* module (Rajagopalan et al., 2006) and both these noncoding loci are regulated by a nutrient/stress-response MYB75 feedback loop (Hsieh et al., 2009; Luo et al., 2012). Our results indicate that high exogenous sucrose treatments cause significant transcriptional reprogramming both at the miRNA and mRNA transcript levels, with evidence for the former causing the latter by AGO effector slicing for several modules in secondary metabolism/phenylpropanoid pathways such as miR828, miR858, and *TAS4*-3′ D4 (-) tasiRNA (Rajagopalan et al., 2006; Luo et al., 2012). We also found miR158/173 and cognate target *Pentatrico Peptide Repeat (PPR*) genes were significantly differentially regulated by high sucrose stress, and several novel modules as targets of miRNAs involved in carbon secondary metabolism such as miR408: *Flavonoid 3′Hydroxylase/F3′H*, miR828:*MYB82*, miR858a/b: *MYBL2*, and primary photosynthetic pigment biosynthesis miR398bc*:*At ORANGE/OR* via analysis of publicly available degradome datasets. Considering the central role of sucrose in cellular homeostasis, this work expands the knowledge of sugar and stress hormone crosstalk regulatory pathways impacting carbon fluxes from primary to antioxidant secondary metabolism.

## Materials and methods

### Plant materials, growth, and sucrose treatments

Homozygous activation-tagged *pap1-D* (ABRC stock CS3884) (Borevitz et al., 2000) and control Col-0 seeds (CS70000) were obtained from the Arabidopsis Biological Resource Center. Seed stocks for uncoupling protein UCP single mutants (*ucp1*, *ucp2*, and *ucp3*) and doubly heterozygous *ucp12*, *ucp13*, and *ucp23* genotypes were the gift of Dr. Ivan Godoy Maia, São Paulo State University, Botucato, Brazil; genotyping and molecular/physiological/phenotypic characterization of homozygous stocks derived from ABRC lines CS874648, SALK_037074, and SALK_123501C (Alonso et al., 2003), respectively, and triple *ucp* mutant *ucp123* genotypes, will be described elsewhere. We used the *ucp* genotypes as additional biological replicates of the sucrose induction effects on *MIRNA* expressions; we did not detect any *ucp* genotype effects on *MIRNA*s per se (see below; Azad, 2022).

For sucrose induction experiments, 3-day-old Arabidopsis mutants (*PAP1-D*, *ucp1*, *ucp2*, *ucp3*, *ucp12*, *ucp23*, *ucp13*, and *ucp123*) and control (Col-0) seedlings were germinated and grown as described (Luo et al., 2012). Following stratification and germination for 3 days, seedlings (~150 seedlings per biological sample) on filter papers in Petri plates containing Murashige and Skoog standard medium (MS medium, one-half strength, control) were transferred to Petri plates containing ½ MS medium plus 200 mM sucrose (6.8% w/v) and allowed to grow at room temperature on a bench under continuous light for 3 days (72h). The other half of seedlings was moved aseptically to ½ MS medium without sucrose, which served as environmental treatment control. After 72h, sucrose-treated and untreated seedlings were harvested by freezing in liquid nitrogen and/or used for various downstream experiments such as ROS assays, anthocyanin quantification, RNA and sRNA extraction, and deep sequencing library preparation.

### Anthocyanin quantification

Anthocyanin quantification of sucrose-treated and untreated control genotypes was done according to the pH differential protocol (Lee et al., 2019). Approximately 100 mg of frozen seedling tissue was pulverized to powder with mortar and pestle in liquid nitrogen, added to 1 mL of extraction buffer (1% [v/v] hydrochloric acid in methanol), and incubated at 4°C overnight. Extracts were centrifuged for 15 min at 15,000 rpm, and supernatant was transferred to a new tube. From there, two solutions were made by adding equal volumes of supernatant to pH 1.0 and 4.5 buffer volume, and absorbance readings were taken at 520 nm and 700 nm for both solutions. Notably, before taking the final readings, several dilutions using pH 1.0 and pH 4.5 buffers were made until absorbance at 520 nm was within the linear range of the spectrophotometer (ThermoScientific Biomate 5). Anthocyanin quantity was expressed as cyanidin-3-glucoside equivalents (mg/L); see **Supplementary Methods File** for formula).

### 3,3′-diaminobenzidine and nitroblue tetrazolium stains for reactive oxygen species accumulation

3,3′-diaminobenzidine (DAB) and nitroblue tetrazolium (NBT) reagents were used to detect hydrogen peroxide (H_2_O_2_) and superoxide anion (O_2_^−.^) species, respectively (Kumar et al., 2014). For DAB staining, Thermo Scientific™ Pierce™ DAB Substrate Kit (#34002) was used according to the manufacturer’s protocol. For NBT staining, Invitrogen™ Nitro blue Tetrazolium Chloride (#N6495) was used. For DAB staining, a 1X solution was made according to the manufacturer protocol and for NBT staining, a 0.2% NBT solution was made in an amber-colored bottle by dissolving 0.1 g NBT in 50 mM sodium phosphate buffer (pH 7.5). Ten to fifteen 6-day olds freshly collected sucrose-treated and untreated seedlings were immersed in 1X DAB or NBT solution and vacuum infiltrated for 5 min and subsequently incubated on a shaker for 6h–8h in the dark. Following incubation, the DAB and NBT solutions were drained off and replaced with a bleaching solution (ethanol:acetic acid:glycerol = 3:1:1) and placed in a boiling water bath (~90°C–95°C setting) for 15 min. The bleaching procedure was repeated once more with fresh bleaching solution and samples were allowed to stand for 30 min. Samples at this stage were stored at 4°C or immediately photographed.

### RNA and small-RNA library preparation and sequencing

Total RNA and sRNAs were extracted from the sucrose-treated and untreated samples (biological duplicates, except sucrose treatment of *pap1-D* and Col-0 had biological triplicates). Approximately 100 mg of the tissue was used for sRNA extraction with miRPremier microRNA isolation kit (Sigma-Aldrich, Saint Louis, MO), or total RNA from 100-mg aliquots extracted with Spectrum Plant Total RNA extraction kit (Sigma-Aldrich) per the manufacturer’s protocols and quantified using Nanodrop (ND-1000 Spectrophotometer; Thermo Fisher Scientific, Waltham, MA). The isolated sRNA was further quantified on Agilent 2100 Bioanalyzer instrument using small-RNA kit (catalogue #-5067-1548), and total RNA was quantified and qualified (RIN > 6.0) using Agilent RNA 6000 Nano Total RNA analysis kit (catalogue #5067-1511) according to manufacturer’s protocols. See **Supplementary Methods File** for library sequencing details.

### Sequence data analyses

Bioinformatics methods for sequence data analysis were as described previously (Sunitha et al., 2019; Mittal et al., 2023), and scripts were provided in **Supplementary Methods File**. In brief, the RNA-seq and sRNA-seq libraries were quality assessed using FastQC v0.11.5 (https://www.bioinformatics.babraham.ac.uk/projects/fastqc/). The sRNA libraries were trimming using fastx_clipper tool of the FASTX toolkit (http://hannonlab.cshl.edu/fastx_toolkit/index.html) and reads with length greater than 18 bp were retained, whereas for RNA-seq data, adapter clipping was performed using Trimmomatic (Bolger et al., 2014). Quality-assured sRNA-seq and RNA-seq reads were subjected to sequential filtration steps to remove structural RNAs mapping to *Arabidopsis thaliana* ribosomal RNAs (rRNAs), transfer RNAs (tRNAs), small nucleolar RNAs (snRNAs), and transposable elements (TEs) (https://ftp.ebi.ac.uk/ensemblgenomes/pub/release-56/plants/fasta/arabidopsis_thaliana/ncrna/, https://www.arabidopsis.org/download_files/Genes/TAIR10_genome_release/TAIR10_transposable_elements/TAIR10_TE.fas) using bowtie-1.1.2 (Langmead and Salzberg, 2012) (**Supplementary Datasets S1**, **S2**). Additional publicly available sRNA data were downloaded from National Center for Biotechnology Information (NCBI), processed and quality assured in order to increase the confidence of ShortStack miRNA identification steps (PRJNA110625, PRJNA634468, PRJNA300285, PRJNA251351, PRJNA316991, PRJNA389307, PRJNA413472, PRJNA415623, PRJNA522058, PRJNA190673, and PRJNA560782). The filtered clean RNA-seq reads were mapped using Kallisto (Bray et al., 2016) to a custom Arabidopsis cDNA reference created by adding all of the representative protein coding genes downloaded from TAIR10 and pri-*MIRNA* sequences downloaded from miRBase (version 22) (Kozomara et al., 2019). The filtered clean sRNA-seq reads were mapped to *Arabidopsis thaliana* TAIR10 genome using ShortStack version 3.8.5 (Johnson et al., 2016) for *de-novo* characterization and quantification of Arabidopsis *MIRNA* loci (Kozomara et al., 2019), explained in detail in **Supplementary Methods File**.

The raw counts generated by Kallisto and ShortStack were utilized as an input for respective differential expression analysis in DESeq2 R package (release 3.14) (Love et al., 2014). A false discovery rate (FDR) multiple-testing approach was applied (Benjamini and Hochberg, 1995) with default 5% FDR as cutoff. Technical replicates were tested for significantly differential effects, and none were found (data not shown). Principal component analysis (PCA) plots were generated using a web platform iDEP version 1.1 (Ge, 2021). A heatmap of the differentially expressed sucrose responsive sRNAs (miRNAs and tasiRNAs; p-adjusted < 0.007) was generated using TBtools (Chen et al., 2020). For the heatmap analysis, only the high-confidence miRNAs were taken into consideration as per (Taylor et al., 2014; Taylor et al., 2017; **Supplementary Dataset S3**). In addition, UpSet plots displaying the number of significantly differentially expressed loci (*p*-adjusted < 0.05) in response to sucrose treatments in control and *pap1-D* genotypes were generated using UpSetR package (Lex et al., 2014). The sequencing runs have been submitted as raw fastq files to NCBI Sequence Read Archive with BioProject accession PRJNA995345.

### Systems analysis of differential expression by Gene Ontology MapMan and PageMan over-representation analysis

To represent the metabolic processes and pathways differentially regulated in response to treatment and genotype(s) as represented by RNA-seq data analysis, genome-wide output from DESeq2 was subjected to MapMan analysis (Schwacke et al., 2019) at *p*-adjusted < 0.05 cutoff. PageMan is an embedded feature of Mapman that uses over-representation analysis to identify functional categories of biological/metabolic processes and pathways that are significantly over- or under-represented (Usadel et al., 2006). Wilcoxon test was performed on the DESeq2 results using PageMan and applying the most stringent parameter setting (3) and sub-setting the differentially expressed genes based on *p*-adjusted cutoff of ≤ 0.05, and the results were displayed as interactive heatmaps for the enriched and depleted functional categories and pathways.

### Small-RNA Northern blot analysis

700 ng of sRNA from the sucrose-treated and untreated seedling samples were used for sRNA Northern blotting as described in (Mittal et al., 2023). Synthetic DNA oligonucleotides (**Supplementary Table S1**) (Sigma-Aldrich, St. Louis, MO) complimentary to specific miRNAs were used as probes after 5´-end-labeling using ^32^P- γATP, 6,000 Ci/mmol, (PerkinElmer). A 22-nt anti–vvi-miR828 probe was a locked nucleic acid oligonucleotide (Exiqon Inc., Woburn, MA). The sRNA blot band relative intensities were quantified using ImageJ and normalized to loading per lane SYBR Gold-stained bands as validation of reproducibility and linear response of signal strengths. 5S rRNA probe signal and SYBR Gold-stained (Thermo Fisher Scientific) 5S and tRNA abundances visualized on gels used for blots were quantified by ImageJ (Schneider et al., 2012) and used as an internal/loading control for normalization of blot test signal strengths. Numerical signal values for sucrose treatment effects are expressed as the ratio of miRNA probe signal to the SYBR Gold-stained total 5S/tRNA band slices, relative to the loading-normalized signal of control untreated signals set to unity.

### miRNA-target mRNA interaction analysis and identification of PHAS loci

Degradome (Gregory et al., 2008; Addo-Quaye et al., 2009; German et al., 2009) datasets for the *Arabidopsis thaliana* were downloaded from NCBI (SRR6041117, SRR6041069, SRR7652712, and SRR7652709) and were subjected to quality control with FastQC v0.11.5 and, if necessary, adaptor removal and trimming with fastx_clipper. The processed reads were then mapped to structural RNAs in *Arabidopsis thaliana*, that is, rRNAs, tRNAs, snRNAs, and TEs and the reads mapping to these structural RNAs were filtered out using bowtie2 (Langmead and Salzberg, 2012). Phased, small-interfering RNA-generating loci (PHAS loci) and their candidate miRNA triggers were identified using filtered degradome, ath-miRNAs (from miRBase22), and sRNA library inputs to PhaseTank software (Guo et al., 2015). Filtered reads were then subjected to CleaveLand4 to predict and identify potential AGO cleavage sites (Addo-Quaye et al., 2009). CleaveLand4 implements Generic Small RNA-Transcriptome Aligner (GSTAr) (https://github.com/MikeAxtell/GSTAr) to calculate duplex parameters on RNA-RNA thermodynamics in addition to sequence-based alignment. Outputs generated by CleaveLand4 for all the publicly available degradome datasets were compiled, and miRNA:target interactions with greater than two independent slicing T-plot evidence sources were subjects for further analysis.

## Results

### Exogenous high sucrose treatment induces anthocyanin and reactive oxygen species production in seedlings

**Figure 1:**
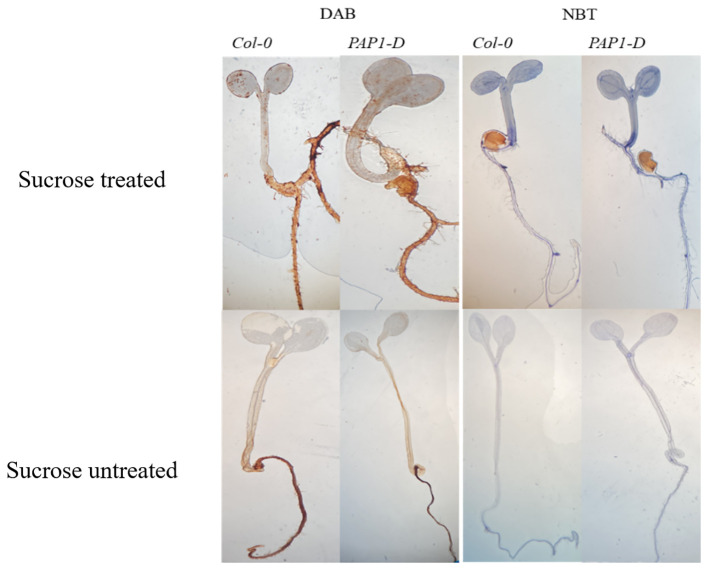
DAB and NBT staining of the sucrose treated and untreated Col-0 and *pap1-D* seedlings shows high sucrose stress treatment results in reactive oxygen species production.

In plants, excess sugar accumulation is associated with the accumulation of anthocyanin pigment, which is a stress biomarker (Couée et al., 2006; Sami et al., 2016; Jeandet et al., 2022) mediated in part by crosstalk with ABA stress hormone (Finkelstein et al., 2002; Luo et al., 2012; Rodrigues et al., 2013), but the molecular mechanisms are not well understood. Exogenous 6.8% (w/v) 200 mM sucrose treatment caused accumulation of anthocyanin in both genotypes, with *pap1-D* genotype having significantly higher anthocyanin accumulation than control Col-0 genotype with or without exogenous sucrose (**Supplementary Figure S1**), confirming the reproducibility of our earlier study (Luo et al., 2012). *pap1-D* genotype constitutively overexpresses *PRODUCTION OF ANTHOCYANIN PIGMENT 1* (*PAP1*)/*MYELOBLASTOSIS PROTEIN 75* (*MYB75*) transcription factor (Borevitz et al., 2000) and, thus, serves as a validated check for exploring genetic interactions with hypothesized sRNA effectors of sugar signaling and homeostasis, because it is targeted by miR828:*TAS4* module (Rajagopalan et al., 2006), and both *MIR828* and *TAS4* locus expressions are regulated by a nutrient stress-response MYB75 feedback loop (Hsieh et al., 2009; Luo et al., 2012). Anthocyanins, by virtue of their antioxidant properties function as ROS scavengers and facilitate cellular ROS homeostasis during abiotic stresses (Naing and Kim, 2021). Previous studies have shown that production of ROS can affect various metabolic as well as physiological processes such as photosynthesis, cell differentiation, cell growth and signaling pathways (Miller et al., 2010; Kärkönen and Kuchitsu, 2015; Ribeiro et al., 2017; Zeng et al., 2017). In addition, superoxide functions as a metabolic signal associated with sugar levels (Román et al., 2021). We measured ROS by chromogenic staining of seedlings subjected to high exogenous sucrose stress. **Figure 1** shows that sucrose stress treatment resulted in increased production ROS in both Col-0 and *pap1-D*, as manifested by DAB and NBT staining for H_2_O_2_ and O_2_^−^, respectively, compared to control untreated seedlings. Thus, the expected increase in ROS in response to high sucrose stress treatments that increase anthocyanin accumulation establishes our experimental system as appropriate for assaying mRNA, miRNA, and tasi-RNA abundance changes in response to exogenous sugar stress.

### Correlation of small-RNA changes to high sucrose treatment

Given the pivotal role of sRNAs in the regulatory networks that govern plant responses to abiotic stresses (Sunkar and Zhu, 2004; Liu et al., 2020; Islam et al., 2022; Mittal et al., 2023) and low concentration (1% w/v) of sugar (Dugas and Bartel, 2008), it is of interest to investigate the impact of high sucrose (200 mM, 6.8% w/v) stress treatment on the seedling sRNAome. The expression of stress-related sRNAs may impact through post-transcriptional gene silencing the capacity to tolerate and adapt to adverse environmental conditions. PCA, as shown in **Supplementary Figure S2A**, of all sRNA-generating clusters across genotypes and sucrose treatment revealed that treatment was the major variable affecting sRNA abundance. PC1 and PC2 pseudo-dimensions correlated significantly with treatment at *p* < 0.001 and accounted for ~35% and ~12% of observed expression variation across samples, respectively. 21 nt sRNA species were more abundant than 24 nt sRNA species in both treated and untreated libraries (**Supplementary Figure S3**), as expected for Arabidopsis at seedling stage (Jung et al., 2015; Tripathi et al., 2019; Aslam et al., 2020; Wu et al., 2021).

### Characterization of high sucrose responsive miRNAs and phasiRNAs

Because most miRNAs and phasiRNAs are 21 nt in size and constitute a high proportion of 21 nt sRNA abundance, but not diversity, associated with biotic stress (Källman et al., 2013), we characterized the differential accumulation of miRNAs and phasiRNAs annotated and quantified by ShortStack (Johnson et al., 2016) from seedlings subjected to high exogenous sucrose treatment. The overall sucrose treatment effect was determined using DESeq2 Wald-Log test (Love et al., 2014) on cluster counts for all genotypes (Col-0, *PAP1-D*, and *ucp1*/*2*/*3*/*1,2*/*1,3*/*2,3*/*1,2,3*) in the design matrix. Because we found no effect of *ucp* genotype or *ucp* genotype-by-sucrose treatment interaction effects on miRNA differential expression (Azad, 2022; **Supplementary Dataset S3**), the above design matrix provided a high degree of biological replication (*n* = 18) for a pure sucrose treatment effect on miRNA differential expressions, paired to genotype samples across biological replicate treatments.

**Figure 2:**
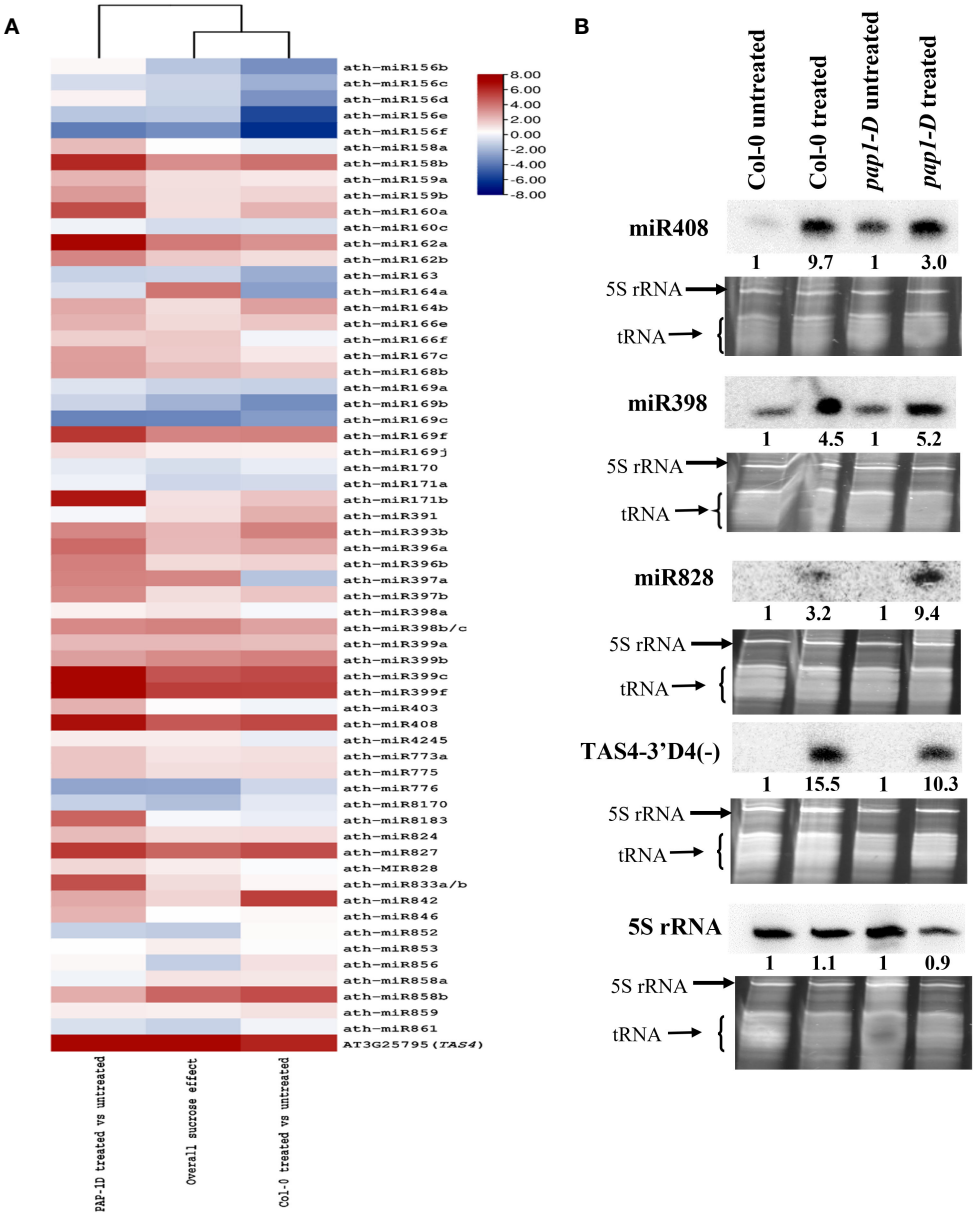
microRNA differential expressions by high sucrose treatment. **(A)** Heatmap clustering of sucrose responsive significantly differentially expressed small RNAs (miRNAs + tasiRNAs) (*padj* < 0.05) in Col-0, *pap1-D* and overall (Col-0 + *pap1-D*) test. **(B)** Small-RNA Northern blot analysis of miRNAs miR398, miR408, miR828, miR828-derived tasiRNA TAS4-3′D4(-), and 5S rRNA in sucrose-treated and untreated Col-0 and *pap1-D* seedling samples. Blot signals were normalized relative to SYBR Gold-stained gel band intensities and numbers calculated relative to untreated controls (set to unity) for fold-effects of sucrose induction.

A comprehensive analysis revealed that miRNAs from 36 high-confidence (Taylor et al., 2014) miRNA families exhibited significant differential expression in response to exogenous sucrose treatment (overall sucrose treatment effect, *p*-adjusted < 0.007), as shown in **Figure 2A**. Several miRNAs have previously been identified as responsive to abiotic stress, specifically nutrient stresses (Liang et al., 2015), and are involved in sucrose signaling (miR156, miR398, and miR408), the phenylpropanoid pathway (miR156, miR828, miR858), response to inorganic phosphate (Pi) (miR399 and miR827), nitrogen homeostasis (miR169), ABA signaling (miR842 and miR169), gibberellin (GA) signaling (miR159), and copper homeostasis (miR398 and miR408) (Dugas and Bartel, 2008; Ren and Tang, 2012; Ma et al., 2015; Meng et al., 2021; Azad et al., 2023). The majority of differentially expressed miRNAs exhibited an upregulation trend, except for the miR156 family, miR163, miR776, and miR8170 (**Figure 2A**). Although the overall trend in expression among members of the miRNA family was consistent, certain miRNA families such as miR164, miR169, and miR397 exhibited notable exceptions as the members of these miRNA families displayed contrasting trends in expression compared to one another (**Figure 2A**).

Previous studies have established the importance of *PAP1*/*MYB75* in mediating sugar (glucose and sucrose) signaling and juvenile to adult phase transition. We also observed a significant (*p* < 0.05) effect of genotype-by-sucrose interaction on the expression of various miRNAs in *PAP1-D* genotype background (**Figure 2A**; **Supplementary Dataset S3**, columns P–R). For example, a positive genotype-by-sucrose interaction was observed for miR156abde, miR162ab, miR169b, miR397ab, and miR399f as accumulation of these miRNA clusters were increased because of sucrose effect in *PAP1-D* genotypic background as compared to overall sucrose treatment and effect of sucrose in Col-0 (control genotype) (**Supplementary Dataset S3**, columns D and G). On the other hand, a negative genotype-by-sucrose interaction was observed for miR391 and miR776 as accumulation of these miRNA clusters were downregulated because of sucrose effect in *pap1-D* genotypic background as compared to overall sucrose treatment and effect of sucrose in Col-0 control genotype (**Supplementary Dataset S3**).

Apart from miRNA, tasiRNAs generated from *TAS* loci are also a major constituent of 21 nt sRNAs. An evolutionarily conserved autoregulatory feedback loop affecting miR828-*TAS4*-*PAP1*/*MYB75* has been shown to play a significant role in the regulation of anthocyanin biosynthesis pathway in response to sucrose treatment (Luo et al., 2012). We also observed that generation of tasiRNAs from *TAS4*, TAS4-3′D4(-) being the most abundant tasiRNA species, were significantly upregulated (*p*-adjusted < 0.001) in response to sucrose treatment (**Figure 2A**, **Supplementary Dataset S4**).

We independently validated our miRNA and siRNA differential expression claims by small-RNA Northern blotting analysis. We selected small RNAs that exhibited significant differential expression in our study and were previously identified as sucrose responsive (Dugas and Bartel, 2008; Luo et al., 2012; Ren and Tang, 2012), namely, miR398, miR408, miR828, and *TAS4*-3′ D4 (-). As expected, based on prior claims and in concordance with our sRNA-seq results (**Supplementary Dataset S3**) we found an over-accumulation for miR408, miR398, miR828, and TAS4-3′ D4(-) showed by increased band intensities in the sucrose-treated samples as compared to untreated samples (**Figure 2B**). In addition, a relatively higher expression for miR408 (~6×) and miR398 (~1.5×) was found in *pap1-D*–untreated samples as compared to Col-0 untreated samples (**Figure 2B**). Although non-significant statistically, our sRNA-seq results reflect the blot-manifested positive genotype-by-sucrose interaction for miR398 and miR408 in the *pap1-D* genotype (**Supplementary Dataset S3**). Interestingly, miR828 and *TAS4*-3′ D4 (-) remained undetectable in untreated control blots, which was consistent with our sRNA-seq findings, as sRNA reads mapping to sRNA clusters corresponding to miR828 and *TAS4* were significantly lower in untreated samples compared to sucrose-treated samples (**Supplementary Dataset S3**, columns BY-DI); **Supplementary Dataset S4**, columns CG–DQ).

### Correlation of transcriptomic changes to high sucrose treatment

To elucidate how high exogenous sucrose treatment influences the transcriptomic landscape of seedlings, we analyzed RNA-seq data generated from the sucrose-treated and untreated samples. PCA, as shown in **Supplementary Figure S2B**, of all expressed transcripts across all nine genotypes and treatment, revealed that sucrose treatment was again the major variable significantly represented in PC1 pseudo-dimension accounting for ~50% of variation, as seen for sRNA-seq data from the same sample. It was apparent there was a batch effect across biological replicates represented in PC2 pseudo-dimension due to technical differences, in particular wide variability in rRNA contamination, and two different sequencing lengths (50 bp and 150 bp) for interrogated biological replicate libraries (**Supplementary Dataset S1**), which directly affected transcriptome read depth and coverage. Notwithstanding, the biological replicates for the genotypes of interest (Col-0 and *pap1-D*) and treatment were paired factors included in the DESeq2 design matrix to address the batch effect in the differential expression by sucrose treatment analysis. Once again *ucp* genotypes were not included in the RNA-seq data analysis since no effect of *ucp* knockout genotypes was observed on miRNA expression across all *ucp* genotypes in the sRNA-seq data (Azad, 2022; **Supplementary Dataset S3**) and RNAseq analysis of *ucp* mutants will be described elsewhere.

### Transcriptional reprogramming by high sucrose treatment

RNA-seq data analysis revealed that 8,106 genes were significantly differentially expressed in response to sucrose treatment for the overall sucrose effect paired across Col-0 and *pap1-D*. Of those 8,106 loci, the sucrose effect in Col-0 manifested as 3,934 DE genes, and the sucrose effect in *pap1-D* genetic background was 4,449 genes, respectively (**Supplementary Figure S4**, **Supplementary Dataset S5**, columns I and L). **Supplementary Figure S4** shows an overlap (3,250) in the significantly differentially expressed genes in response to sucrose treatment across all three abovementioned tests, and a ~40% increase in the intersection of *pap1-D* sucrose effect versus overall sucrose effect (1,189) compared to Col-0 versus overall sucrose effect (675) (**Supplementary Figure S4**). The difference in gene numbers may reflect in part a genotype-by-treatment interaction of 187 genes (**Supplementary Dataset S5**, column N) with an observed *p* < 0.05 for *pap1-D* as a check on a hypothesized genotype effect, since PAP1 is a TF regulating secondary metabolite anthocyanin biosynthesis (Borevitz et al., 2000). Approximately 50% of the mentioned 187 loci showed a significant genotype by sucrose interaction at *p* < 0.05 in *pap1-D* genotypic background (**Supplementary Dataset S5**, column Q) and as expected, the effectors of flavonoid biosynthesis pathway, members of MBW (MYBs, bHLHs, and WD40s) complex (see below), were found to be affected by both sucrose treatment as well as *PAP1* overexpression (*pap1-D* genotypic effect).

**Figure 3:**
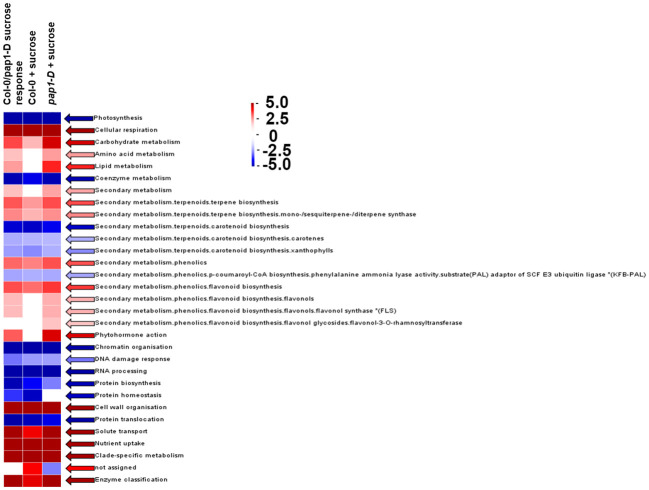
PageMan Gene Ontology global transcriptional analysis of significantly over-represented/enriched biological process bins in response to sucrose in Col-0, *pap1-D*, and overall (Col-0 + *pap1-D*) using the Wilcoxon Rank Sum Over Representation Analysis cutoff setting = 3 (most conservative/stringent) and Benjamini-Hochberg multiple testing correction algorithm mapped to 33,000 annotated Arabidopsis genes. Genome-wide over-represented bins are aligned with Kyoto Encyclopedia of Genes and Genomes Orthology for proteome function terms highlighted in red (upregulated overall) and blue (downregulated overall), respectively. The test analyzes the median fold change within the ontological group against the median fold change of all genes not in that ontological group. The scale shown is for Z test scores which approximate standard deviations from median.

To identify the differentially regulated metabolic pathways in response to high sugar stress in Col-0 and *pap1-D* genotypes, output from DESeq2 for RNAseq was subjected to genome-wide enrichment analysis using PageMan, an embedded MapMan feature (Usadel et al., 2009; Schwacke et al., 2019). For overall sucrose treatment, the over-represented differential expression bins corresponding to photosynthesis, co-enzyme metabolism, chromatin organization, DNA damage response, RNA processing, protein homeostasis, and translocation significantly over-represented for the genes downregulated in response to high sucrose treatment as shown in **Figure 3**. Conversely, the bins corresponding to cellular respiration, carbohydrate metabolism, secondary metabolism, phytohormone action, cell wall organization, solute transport, and nutrient uptake were significantly over-represented for the genes upregulated in response to sucrose treatment (**Figure 3**).

All of the aforementioned bins showed comparable trends for under- or over-representation in response to sucrose treatment in Col-0 and *pap1-D* (**Figure 3**). Foyer et al. (2012) have shown that application of exogenous sucrose represses photosynthetic genes via retrograde signaling from plastids to nucleus. Since oxygen and glucose are both needed for cellular respiration, an increase in sugar concentration could result in a decrease in oxygen concentration resulting in inhibition of cellular respiration. An over-representation of upregulated genes mapped to cellular respiration bin can be speculated as a homeostatic response involving mitochondrial retrograde signaling (Barreto et al., 2022).

**Figure 4:**
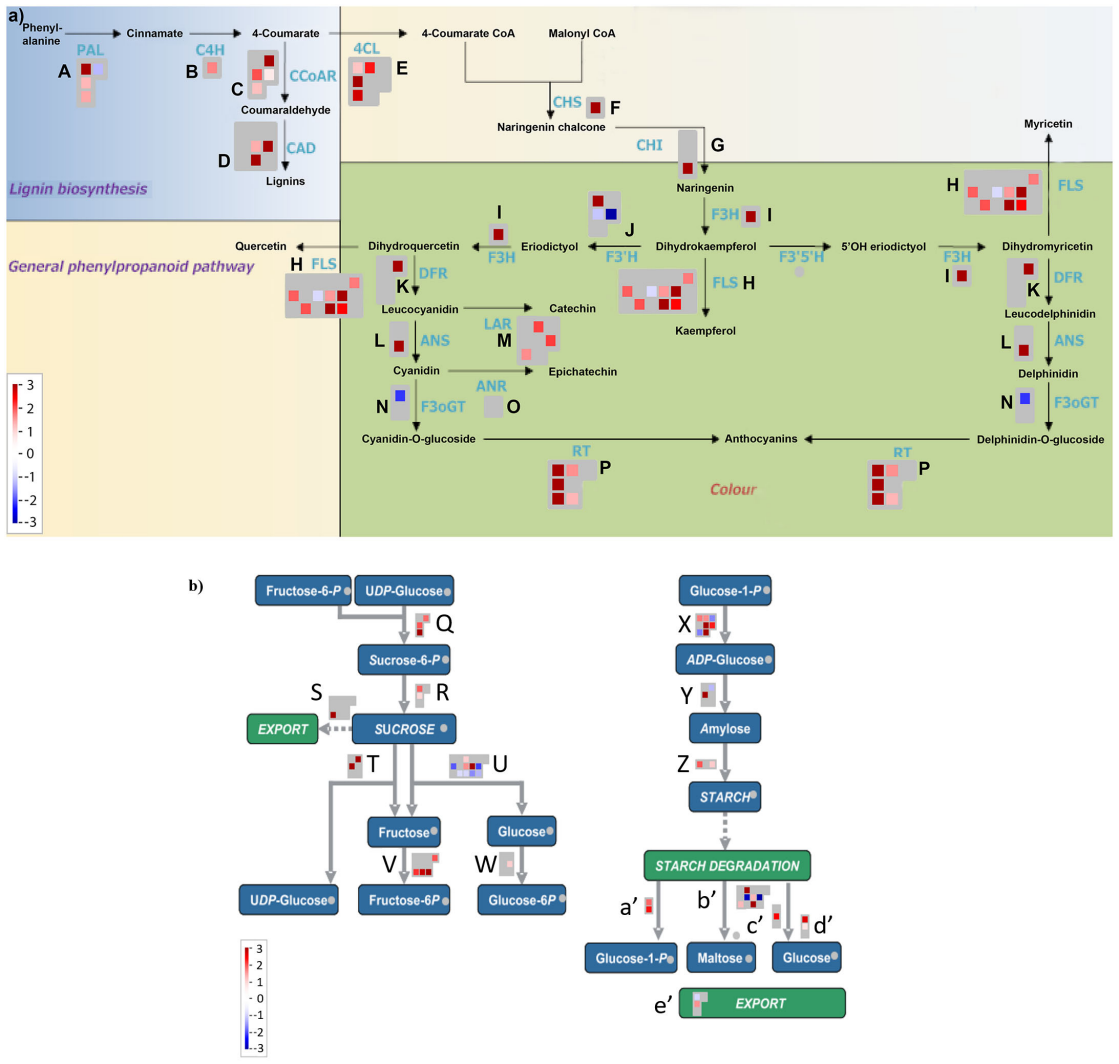
**(A)** MapMan flavonoid pathway analysis of differentially expressed genes from Overall Sucrose versus non-sucrose control comparison. **(B)** MapMan sucrose-starch metabolism pathway analysis of differentially expressed genes from overall sucrose versus non-sucrose comparison. See Supplementary Dataset S6 for gene names and color-coded expression numerics associated with biochemical steps/arrows (blue font in panel A, abbreviated enzymes), labeled alphabetically.

**Figure 5:**
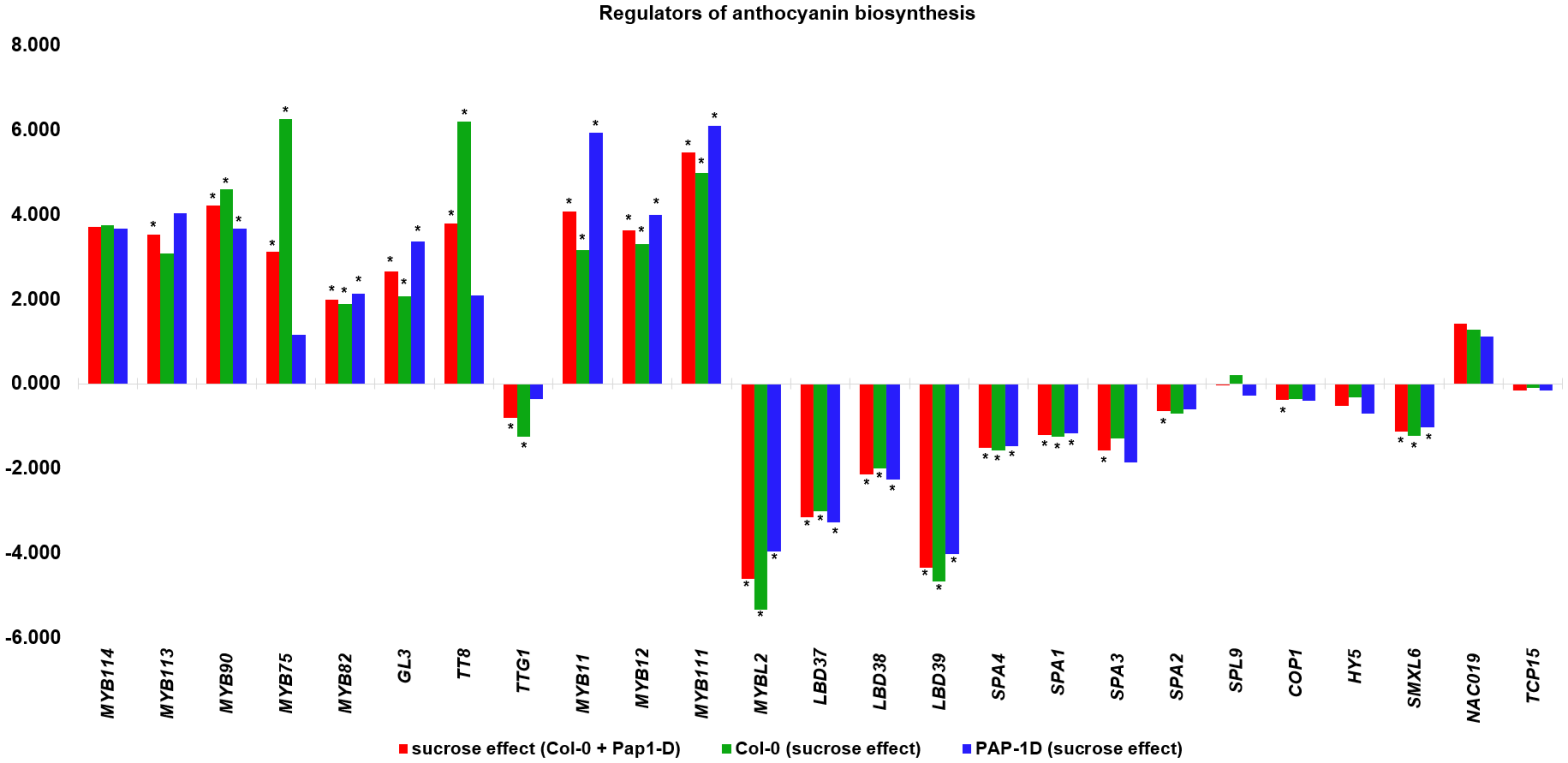
Differential expression (Log_2_FC) of positive (*MYB114*-*MYB111*) and negative regulators (*MYBL2*-*TCP15*) of anthocyanin pathway from Overall sucrose versus non-sucrose control comparison. * indicates *padj* < 0.05.

Previous studies reported the effect of varying sucrose concentrations on the modification of specific flavonoids in various plant species (Loreti et al., 2008; Morkunas et al., 2011; Luo et al., 2012; Kim et al., 2020; Li et al., 2020a; Peng et al., 2020; Lv et al., 2022). Along the same lines, our MapMan results showed that high sucrose treatment activated the whole phenylpropanoid pathway genes leading to enhanced flavonoid and anthocyanin biosynthesis (**Figure 4A**), which is consistent with metabolic flux regulation described for Arabidopsis as a synergy between the anthocyanin biosynthetic and RDR6/SGS3/DCL4 tasiRNA pathways (Jiang et al., 2020). Upon further examination of the flavonoid biosynthesis bin, we found that expression of several known activators of anthocyanin biosynthesis, namely, *MYB75*, *MYB82*, *MYB90*, *MYB113*, *MYB114*, *GL3, TT8*, *MYB11*, *MYB12*, and *MYB111* except *TTG1* were found to be upregulated, whereas expression of the known repressors of anthocyanin biosynthesis, that is, *MYBL2*, *LBD37*, *LBD38*, *LBD39*, *SPA1*, *SPA2*, *SPA3*, *SPA4*, *COP1*, *HY5*, and *SMXL6* were found to be downregulated in response to high sucrose stress (**Figure 5**). Exogenous sucrose can have an impact on the processes of starch synthesis, mobilization, and distribution. Furthermore, it may also influence the equilibrium between the biosynthesis and degradation of starch (Fernie et al., 2002). Our MapMan results also indicated high sucrose resulted in increased expression of genes involved in starch biosynthesis. Expression of genes like sucrose synthases (*SUS1*-*6*), glucose-1-phosphate adenylytransferases (*APL3*/*4*), starch synthases, and starch branching enzymes involved in starch biosynthesis pathway were upregulated in response to high sucrose treatment (**Figure 4B**; **Supplementary Dataset S6**). In addition, genes involved in starch degradation and mobilization like β-amylases (BAM2/5) and α-amylases (AMY1/2) were upregulated (**Figure 4B**; **Supplementary Dataset S6**).

### Expression analysis of cognate target mRNAs for differentially expressed miRNA/tasiRNAs in response to sucrose treatment

To investigate the functional effects of differentially expressed miRNAs in response to high sucrose, it is necessary to first identify their targets, as plant miRNAs primarily suppress the expression of their target genes by programming AGO-mediated slicing of mRNAs rather than translational repression (Arribas-Hernández et al., 2016). In addition to a thorough literature review, publicly available Arabidopsis degradome datasets from the seedling stage of development (the tissue studied here, unless otherwise noted) were analyzed using CleaveLand4 (Addo-Quaye et al., 2009) to identify known canonical as well as novel miRNA slicing events. Validated targets were quantified by RNAseq data from the same samples for which miRNA differential expression was characterized (**Figure 2**) to test for correlations of inferred miRNA activities on target mRNAs (**Table 1**). We observed miR163:SABATH family (*PXMT1*, *FAMT*, *FAMT-L*, and *AT5G38100*), miR164:NACs (*NAC1L*, *NAC80*, and *NAC92*), miR167:*ARF8*, miR169:*NF-YA* family (*NF-YA8*, *NF-YA5*, *NF-YA2*, and *NF-YA10*; non-canonical *JAZ4*), miR393:*TIR1*, miR396:GRFs (*GRF4* and *GRF7*), miR398:targets (*AtCCS*, *AtBCB*, *AT3G15640*, and *AT5G14550*), miR408:targets (*UCC2* and *PAA2*), miR827:*BAH1* and miR858:*MYBL2* modules were significantly mis-regulated by high sucrose treatment in an anti-concordant manner to the DE miRNAs, supporting the hypothesis that these DE miRNAs in response to high sucrose cause DE of cognate miRNA target mRNAs.

**Table 1:** Expression of sucrose responsive miRNAs (*p*-adjusted < 0.05, this study), their putative functions, and the target mRNA expression along with expression profile in response to various nutrient deficiencies (Liang et al., 2015).

		Treatment											
sRNA family	Family members	Exogenous sucrose (+C, overall)	(-C)	(-N)	(-Pi)	(-S)	Target	Annotation	Log_2_FC	pvalue	*padj*	Relationship with the targeting sRNA	Potential roles
miR156	b/c/d/e/f	down	up	up	up		AT2G33810.1	SPL3, SQUAMOSA PROMOTER BINDING PROTEIN-LIKE 3	-3.106	0.000	0.000	concordant	juvenile-to-adult phase transition (Ronemus et al., 2006; Wang et al., 2009; Cui et al., 2014; Xu et al., 2016)
							AT1G27370.1	SPL10, SQUAMOSA PROMOTER BINDING PROTEIN-LIKE 10	-0.833	0.000	0.002	concordant	
							AT5G43270.2	SPL2, SQUAMOSA PROMOTER BINDING PROTEIN-LIKE 2	-0.957	0.001	0.003	concordant	
miR158	b	up	down	down			AT1G64100.1	pentatricopeptide (PPR) repeat-containing protein	2.151	0.001	0.006	concordant	abiotic stress tolerance (Rhoades et al., 2002; Zhang et al., 2011; Liang et al., 2012)
							AT2G03220.1	FUCOSYLTRANSFERASE 1, ATFT1, ATFUT1, FT1, FUCOSYLTRANSFERASE 1, FUT1, MUR2, MURUS 2	0.730	0.000	0.000	concordant	
miR159	a/b	up	down				AT4G26930.1	ATMYB97, MYB DOMAIN PROTEIN 97, MYB97	2.256	0.001	0.004	concordant	Ather development and flowering time regulation (Reyes and Chua, 2007; Millar et al., 2019)
miR163		down	up		up		AT1G66700.1	SABATH FAMILY PARAXANTHINE METHYL TRANSFERASE; PXMT1	5.693	0.000	0.000	anti-concordant	abiotic and biotic stress response (Allen et al., 2004; Ng et al., 2011; Chow and Ng, 2017)
							AT3G44860.1	FAMT, FARNESOIC ACID CARBOXYL-O-METHYLTRANSFERASE	3.589	0.000	0.000	anti-concordant	
							AT5G38100.1	SABATH family methyltransferase	2.191	0.000	0.000	anti-concordant	
							AT3G44870.1	FAMT-L, FARNESOIC ACID METHYL TRANSFERASE-LIKE	2.053	0.000	0.000	anti-concordant	
miR164	a/b	up	down		up	up	AT3G12977.1	NAC1 LIKE TRANSCRIPTION FACTOR, NAC1L	-2.483	0.000	0.000	anti-concordant	growth, development, response to biotic and abiotic stresses (Raman et al., 2008; Koyama et al., 2010; Fang et al., 2014)
							AT5G39610.1	ANAC092, ARABIDOPSIS NAC DOMAIN CONTAINING PROTEIN 92	-1.603	0.002	0.007	anti-concordant	
							AT5G07680.1	NAC DOMAIN CONTAINING PROTEIN 80, NAC080	-0.637	0.004	0.015	anti-concordant	
							AT5G61430.1	NAC DOMAIN CONTAINING PROTEIN 100, NAC100	1.429	0.006	0.021	concordant	
miR167	c	up	down	up	up	down	AT5G37020.1	ARF8, ATARF8, AUXIN RESPONSE FACTOR 8	-0.615	0.001	0.004	anti-concordant	auxin signaling, flower development, and root development (Wu et al., 2006; Liu X. et al., 2021)
							AT1G51760.1	IAA-ALANINE RESISTANT 3, IAR3	2.014	0.000	0.000	concordant	(Kinoshita et al., 2012)
miR169	a/b/c	down	up	down	down	down	AT1G17590.1	NF-YA8, NUCLEAR FACTOR Y, SUBUNIT A8	1.720	0.000	0.003	anti-concordant	ABA signaling, nitrogen homeostasis (Ronemus et al., 2006; Zhao et al., 2011; Sorin et al., 2014; Xu et al., 2014; Li J. et al., 2021)
							AT1G54160.1	NF-YA5, NUCLEAR FACTOR Y A5	1.842	0.011	0.044	anti-concordant	
							AT3G05690.1	NF-YA2, NUCLEAR FACTOR Y, SUBUNIT A2	1.711	0.000	0.001	anti-concordant	
							AT1G48500.1	ATJAZ4, JASMONATE-ZIM-DOMAIN PROTEIN 4	1.377	0.011	0.011	anti-concordant	(Karlova et al., 2013; Gyula et al., 2018)
							AT5G06510.1	NF-YA10, NUCLEAR FACTOR Y, SUBUNIT A10	3.166	0.006	0.028	anti-concordant	
miR393	b	up	down		up		AT3G62980.1	ATTIR1, TIR1, TRANSPORT INHIBITOR RESPONSE 1	-1.088	0.001	0.004	anti-concordant	auxin signaling (Ronemus et al., 2006; Si-Ammour et al., 2011; Wang et al., 2018)
miR396	a/b	up	down		down		AT3G52910.1	ATGRF4, GRF4, GROWTH-REGULATING FACTOR 4	2.063	0.002	0.008	anti-concordant	cell proliferation (Rodriguez et al., 2010; Szczygieł-Sommer and Gaj, 2019)
							AT5G53660.1	ATGRF7, GRF7, GROWTH-REGULATING FACTOR 7	1.849	0.007	0.025	anti-concordant	
miR397	a/b	up	down	down	down	down	AT2G29130.1	ATLAC2, LAC2, LACCASE 2	-1.676	0.011	0.035	concordant	lignin accumulation and stress tolerance (Lu et al., 2013; Li et al., 2019)
miR398	a/b/c	up	down	down	down	down	AT2G28190.1	ATSOD2, COPPER/ZINC SUPEROXIDE DISMUTASE 2, CSD2	-1.404	0.000	0.000	concordant	copper starvation response; ROS homeostasis (Sunkar et al., 2006; Beauclair et al., 2010)
							AT5G14550.1	Core-2/I-branching beta-1,6-N-acetylglucosaminyl transferase family protein	0.882	0.003	0.011	anti-concordant	(Sunkar et al., 2004)
							AT3G15640.1	Rubredoxin-like superfamily protein	0.717	0.004	0.014	anti-concordant	
							AT1G12520.1	ATCCS, COPPER CHAPERONE FOR SOD1	-1.810	0.000	0.000	anti-concordant	
							AT5G20230.1	ATBCB, BLUE COPPER BINDING PROTEIN	-3.537	0.000	0.000	anti-concordant	
	miR398b*						AT5G61670.1	AtORANGE; Cysteine-rich zinc finger; DnaJ-like	-1.500	0.000	0.000	anti-concordant	this work; novel non-canonical target; chromoplast development (Yazdani et al., 2019)
miR399	a/b/c/f	up	down	down	up	down	AT3G09922.1	IPS1; induced by phosphate starvation1	9.672	0.000	0.000	concordant	nutrient recycling; Pi uptake and translocation (Kim et al., 2011)
miR408		up	down	down	up	down	AT5G21930.1	P-type ATPase of Arabidopsis 2, PAA2	-3.329	0.000	0.000	anti-concordant	responds to the availability of copper, iron homeostasis, oxidative stress response, regulation of lignin biosynthesis also controls various aspects plant growth and development (Chorostecki et al., 2012; Liang et al., 2012; Ma et al., 2015; Li et al., 2019; Gao et al., 2022).
							AT2G44790.1	UCC2, UCLACYANIN 2	-1.000	0.000	0.000	anti-concordant	
							AT3G51240.1	F3’H; flavanone 3-hydroxylase	4.357	0.000	0.000	concordant	this work; novel non-canonical target; anthocyanin biosynthesis (Plotnikova et al., 2019; Liu J. et al., 2021)
							AT5G05390.1	Laccase 12	2.417	0.000	0.001	concordant	
							AT5G07130.1	Laccase 13	2.090	0.001	0.003	concordant	
							AT2G02850.1	ARPN, PLANTACYANIN	0.968	0.007	0.024	concordant	
							AT2G47020.1	Peptide chain release factor 1	1.032	0.000	0.000	concordant	(Sunkar et al., 2004)
miR773	a	up	down	up			AT4G14140.2	DNA methyltransferase 2	-2.896	0.000	0.000	anti-concordant	biotic stress response (Li et al., 2010; Zhang et al., 2011)
miR827		up	down	down	up	down	AT1G02860.1	BAH1, BENZOIC ACID HYPERSENSITIVE 1, NITROGEN LIMITATION ADAPTATION, NLA, SYG1	-1.664	0.000	0.000	anti-concordant	nutrient recycling; P_i_ uptake and translocation, plant-pathogen interaction (Hsieh et al., 2009; Lin et al., 2010; Liang et al., 2012)
							AT1G63010.5	PHT5;1, VACUOLAR PHOSPHATE TRANSPORTER 1, VPT1	0.880	0.001	0.006	concordant	
miR828		up			up		AT3G25795.1	TAS4, TRANS ACTING SIRNA 4	5.339	0.000	0.000	concordant	anthocyanin biosynthesis (Rajagopalan et al., 2006; Guan et al., 2014; Tirumalai et al., 2019; Zhang et al., 2020)
							AT5G52600.1	ATMYB82, MYB DOMAIN PROTEIN 82, MYB82	1.990	0.000	0.000	concordant	This work
							AT1G56650.1	ATMYB75, PAP1	3.125	0.001	0.003	concordant	
							AT1G66390.1	ATMYB90, PAP2	4.210	0.000	0.000	concordant	
							AT1G66370.1	ATMYB113, MYB DOMAIN PROTEIN 113, MYB113	3.519	0.010	0.034	concordant	
miR842		up					AT5G38550.1	Jacalin lectin family protein gene	2.904	0.000	0.000	concordant	(De Felippes et al., 2008; Jia and Rock, 2013)
miR856		down					AT5G41610.1	CATION/H+ EXCHANGER 18, ATCHX18	2.131	0.000	0.000	anti-concordant	Na+/H+ antiporter family (Fahlgren et al., 2007)
miR858	a/b	up					AT2G47460.1	ATMYB12, MYB DOMAIN PROTEIN 12	3.625	0.000	0.000	concordant	involved in phenylpropanoid pathway and plant development (Fahlgren et al., 2007; Guan et al., 2014; Tirumalai et al., 2019)
							AT5G49330.1	ARABIDOPSIS MYB DOMAIN PROTEIN 111, ATMYB111	5.469	0.000	0.000	concordant	
							AT1G06180.1	ATMYB13, ATMYBLFGN, MYB DOMAIN PROTEIN 13, MYB13	1.419	0.000	0.000	concordant	
							AT3G62610.1	ATMYB11, MYB DOMAIN PROTEIN 11	4.078	0.000	0.000	concordant	
							AT1G71030.1	MYB-LIKE 2, ATMYBL2	-4.619	0.000	0.000	anti-concordant	this work
TAS4-3’D4(-)		up			up		AT1G56650.1	MYB75, PAP1-D, PRODUCTION OF ANTHOCYANIN PIGMENT 1	3.125	0.001	0.003	concordant	anthocyanin biosynthesis (Rajagopalan et al., 2006; Hsieh et al., 2009; Luo et al., 2012)
							AT1G66390.1	ATMYB90, MYB DOMAIN PROTEIN 90, PAP2, PRODUCTION OF ANTHOCYANIN PIGMENT 2	4.210	0.000	0.000	concordant	
							AT1G66370.1	ATMYB113, MYB DOMAIN PROTEIN 113, MYB113	3.519	0.010	0.034	concordant	

**Figure 6:**
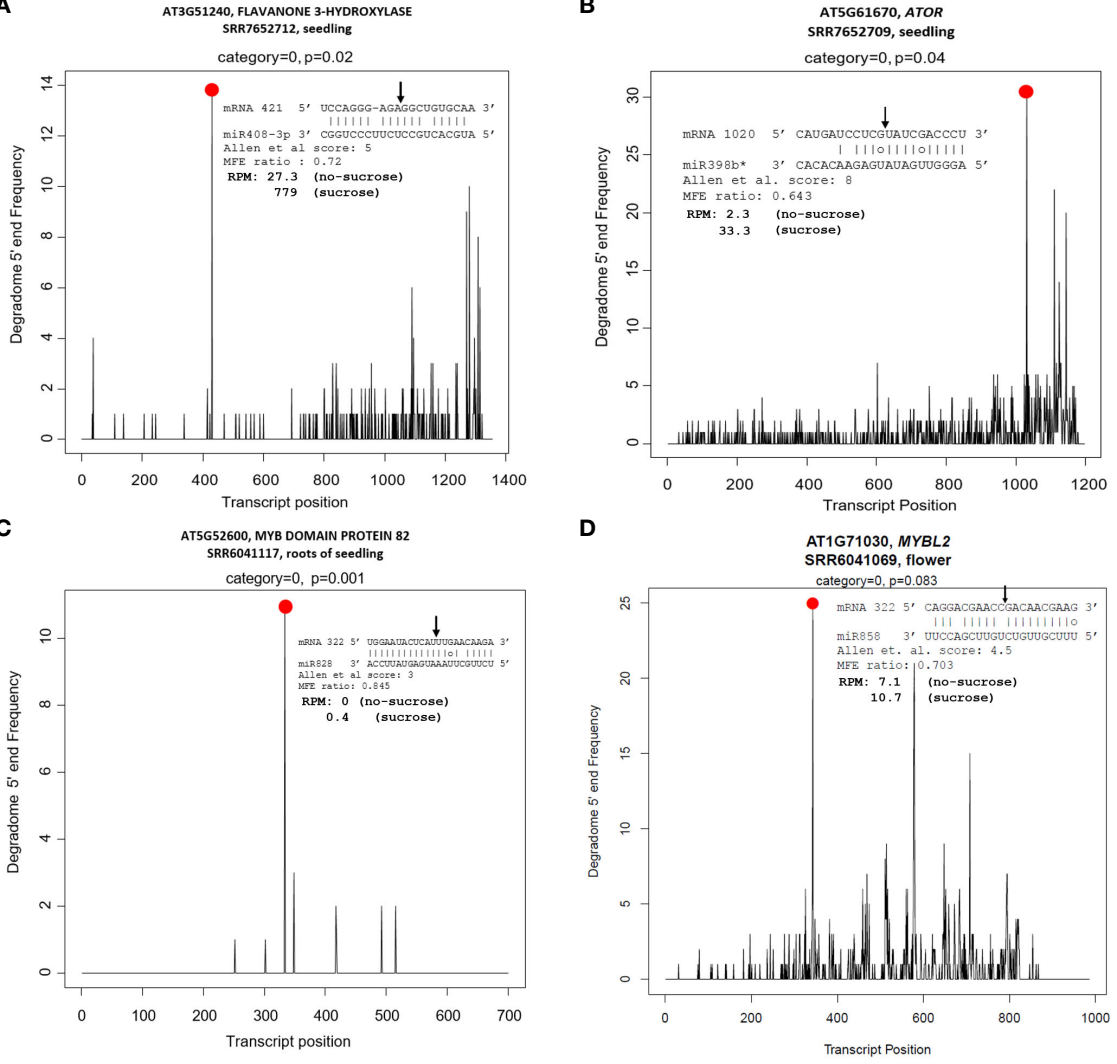
Tissue-specific degradome T plots of canonical and non-canonical candidate miRNA targets related functionally to sucrose stress response pathways. **(A)** miR408 target *F3′H*. **(B)** miR398* target *AtOR*. **(C)** miR828 target *MYB82*. **(D)** miR858 target *MYBL2*. Slicing is at 10th nucleotide from 5’’ end of miRNA (arrow). Black lines are degradome reads; red dot represents claimed slicing degradome reads abundance.

In contrast, miR156:*SPL*s (*SPL2*, *SPL3*, and *SPL10*), miR158:targets (*AT1G64100* and *AtFUT1*), miR159:*MYB97*, miR397:*AtLAC2*, miR398:*CSD2*, miR399:*IPS1*, miR408:targets (*LAC12*, *LAC13*, *ARPN*, and *AT2G47020*), miR827:*VPT1*, miR828:targets (*TAS4*, *MYB82*, and *MYB113*), miR858:*MYB*s (*MYB11*, *MYB12*, and *MYB111*), and *TAS4* tasiRNA TAS4-3′D4(-):*MYB*s (*MYB75*, *MYB90*, and *MYB113*) were mis-regulated in a concordant manner with their miRNA effector DE (**Table 1**), suggesting molecular mechanisms may be involved other than observed miRNA abundances as proxy for inferred AGO slicing activities. We also show clear degradome evidences for miR828 and miR858 directing *MYB82* and *MYBL2* slicing in seedling roots, or flowers, respectively (**Figures 6C, D**). *MYB82* is a predicted yet unvalidated target for miR828 known to play a significant role in the anthocyanin biosynthesis pathway in *Arabidopsis*. Yang et al. (2013b) showed a decrease of *MYB82* transcript level in miR828 overexpression line. Recently, miR828:*MYB82* module has been shown to play a significant role in anthocyanin biosynthesis pathway in response to light stress in *B. rapa* (Zhou et al., 2020). Similarly, we also show that miR828:*MYB82* module may also be involved in anthocyanin biosynthesis in response to sucrose treatment (**Figure 6C**). Previous study shows that miR858a enhances anthocyanin biosynthesis in *Arabidopsis* seedlings via translational repression of *MYBL2*, a negative regulator of anthocyanin biosynthetic pathway (Wang et al., 2016). However, our degradome analysis also found miR858-mediated post-transcriptional slicing evidences for *MYBL2* target transcripts, suggesting a canonical mechanism, at least in flowers of post-transcriptional control of *MYBL2* expression via miR858 (**Figure 6D**), as is known for the majority of miRNA:target modules in plants (Arribas-Hernández et al., 2016).

The majority of the miRNAs in the aforementioned sugar-deranged miRNA:target modules are also known to be differentially expressed in response to macro nutrient starvation (carbon, nitrogen, and Pi) for miR399 and miR827 and micro-nutrient starvation for elements sulfur (miR395) and copper (miR397, miR398, and miR408) (**Table 1**). Furthermore, previous studies in *Arabidopsis* have shown that alamethicin treatment induces miR163 accumulation, implying a role in defense response pathways because alamethicin is a channel-forming fungal peptide antibiotic (Chow and Ng, 2017). This study also claimed increased resistance to *Pseudomonas syringae* in *mir163* mutants, demonstrating a role for miR163 in defense response. miR164: *NAC* and miR169:*NF-YA* modules are known to play important roles in biotic and abiotic stress responses in both monocots and dicots (Fang et al., 2014; Luan et al., 2014; Kushawaha et al., 2019; Li J. et al., 2021). All of these key miRNA:target modules, which have been shown to be mis-regulated in response to abiotic stresses such as nutrient stress (**Table 1**) (Liang et al., 2015), are mis-regulated in response to sucrose treatment. All of the miRNA/siRNA-target modules known to function in phenylpropanoid flavonoid secondary metabolism and anthocyanin biosynthesis showed significant concordant upregulations of the target *MYB* TFs (except *MYBL2*). With exogenously supplied high sucrose, it is hypothesized signal transduction results in increased activity of phenylpropanoid pathway leading to increased flavonol and anthocyanin formation (**Figure 4A**; **Supplementary Figure S4**). A concordant increase in the miR828 and possibly miR858 accumulation initiates a homeostatic feedback response (Hsieh et al., 2009; Luo et al., 2012) to keep the enhanced production of flavonol and anthocyanins in check.

In addition to the known “canonical” miRNA:target modules, we also uncovered two non-canonical miRNA modules in seedlings that target carbon metabolism-relevant novel mRNAs subject to AGO slicing unrelated structurally to the miRNA cognate family genes, specifically miR408:*F3′H* and miR398b/c*:*OR* (**Figures 6A, B**). With an Allen score of 5 and maximum free energy (MFE) ratio of 0.72, miR408 was shown to slice the early pathway anthocyanin biosynthetic gene *F3′H* at nt position 583 for splice variant *AT3G51240.1* and nt 421 for *AT3G51240.2*. For miR398b/c*: *OR/At5g61670* module, an Allen score of 8 and low MFE ratio of 0.64 is shown functional for the slicing of the *OR* mRNA at nt 1031. *F3’H* is coordinately expressed with *chalcone synthase* and *chalcone isomerases* and expressed in concordant fashion as miR408 (upregulated), as observed for other known canonical target *MYB*s involved in flavonol and anthocyanin biosynthesis pathways (**Table 1**). *Arabidopsis OR* encodes a close homolog of the cauliflower *OR* (Orange) DnaJ cysteine-rich zinc-binding domain protein which functions as a molecular chaperone by interacting directly with the Phytoene Synthase protein and is a positive post-translational regulator of Phytoene Synthase expression (Lu et al., 2006; Zhou et al., 2015; Sun et al., 2022). The PageMan bin for carotenoid biosynthesis, a sub-bin of the secondary metabolism bin, was found to be over-represented for differentially downregulated genes in response to sucrose treatment (**Figure 3**). Increased sucrose availability and abiotic stresses could potentially induce the carotenoid biosynthetic pathways, leading to higher carotenoid production (Uarrota et al., 2018; Choi et al., 2019). Contrary to that, we found that the expression of *AtOR* significantly downregulated in response to sucrose treatment. Our observation supports the idea that, in addition to presence and absence of a carbon source, other factors, like stage of plant development (Klepikova et al., 2016), or participation of sRNAs including antisense transcript target mimics of *MIR398* (Li et al., 2020b), may indirectly regulate the carotenoid biosynthesis pathway. Furthermore, evidence that *OR* is a bona fide target of miR398*, whose significant accumulation in response to high sucrose treatment (**Figure 2**, **Table 1**) likely causes the observed novel slicing and thus reduction in gene expression is one piece of the puzzle that may mediate carbon flux shift from primary to secondary metabolism.

## Discussion

Sucrose metabolism is tightly regulated in plants. Altered sucrose metabolism and transport can regulate essential cellular pathways such as plant defense response, ROS production (**Figure 1**) and signaling, mRNA transcription, and translation. How miRNAs are involved in myriad responses to excess sucrose is the subject of our study; in the first instance we explore links uncovered between miRNAs and ROS. A recent report (Xu et al., 2023) is consistent with the notion that miRNA biogenesis can transduce a signal mediating mitochondrial ROS homeostasis in Arabidopsis, where authors showed genetically that a miR400 target *Pentatricopeptide Repeat Protein1* is a positive regulator of cadmium toxic stress by inducing ROS accumulation and promoting RNA editing of the mitochondrial ABC transporter gene *ccmB* involved in cytochrome c biogenesis. Although miR400 was not significantly upregulated in our experiment (**Supplementary Dataset S3**), two of three other miRNAs that also target *PPR*s, miR158 and miR173 (via *TAS1/2*, **Supplementary Dataset S5**; concordantly and significantly upregulated at mature tasiRNA level, as hypothesized, **Supplementary Dataset S4**) were significantly (**Table 1**, for miR158b) or nearly significantly upregulated for miR158a and miR173 (**Supplementary Dataset S3**). A similar result was claimed (Huo et al., 2015) for miR158b down and non-canonical predicted target *fucosyl transferase1/At2g03220/FT1* concordantly down in response to dark-induced senescence, a process of carbon re-allocation. Consistent with that result, we observe concordant changes but in the opposite direction in response to high sucrose stress: both miR158 and *PPR/AT1G64100* target (Rhoades et al., 2002) plus *FT1* target were concordantly upregulated (**Table 1**, **Supplementary Dataset S6**). On the other hand, we observed the majority of validated *PPR* targets of miR173 and miR161 (which manifested non-significant trend of upregulation in response to sucrose; **Supplementary Dataset S3**), namely, *At1g62910, At1g63130, At1g62930, At1g63400, At1g63150*, including PHAS loci *AT1G62914, AT1G63130*, and *AT1G63400* (**Supplementary Dataset S4**) (Howell et al., 2007), were significantly anti-concordantly downregulated by sucrose (**Supplementary Dataset S6**), supporting a hypothesized function in ROS regulation.

In plants, soluble sugars and anthocyanin pathways can synergistically function to detoxify the excess ROS generated during stress conditions (Couée et al., 2006; Van den Ende and Valluru, 2009). In our study, we show that high sucrose treatment results in increased staining for ROS (**Figure 1**) associated with higher accumulation of antioxidant anthocyanins (**Supplementary Figure S1**). Since sucrose serves as a signaling molecule, we hypothesize that a higher accumulation of ROS leads to the activation of antioxidant anthocyanin pathway to ameliorate the plants stress. Are sRNAs a missing link that could mediate/facilitate the sucrose induction of the whole phenylpropanoid pathway (**Figure 4A**) and/or other pathways intersecting plant development and stress responses where sRNAs are known effectors? In accordance to that, an evolutionarily conserved autoregulatory feedback loop involving miR828-*TAS4*: *MYBs* fine tunes the anthocyanin accumulation in response to Pi deficiency and exogenous sucrose stimuli (Hsieh et al., 2009; Luo et al., 2012). We confirmed that miR828 and TAS4-3′D4(-) were upregulated in response to sucrose and relatively highly accumulated in *pap1-D* following sucrose treatment as compared to Col-0 (**Figure 2B**). We were unable to detect a signal for TAS4-3′D4(-) in *pap1-D* untreated samples, contrary to what we previously demonstrated (Luo et al., 2012), which could be due to the prolonged experimental duration (72h), whereas (Luo et al., 2012) demonstrated in a time-course experiment a maximum accumulation of TAS4-3′D4(-) at 12h of sucrose which was reduced by half after 24h. We also showed that TAS4-3′D4(-) as well as its cognate mRNA targets *MYB75*, *MYB90*, and *MYB113*, components of MYB-bHLH-WD40 ternary complex and positive regulators of the late anthocyanin biosynthetic genes, were concordantly upregulated in response to sucrose treatment (**Table 1**). A similar auto-regulatory loop involving miR828-*TAS4*-*MYBA6*/*A7*/*A5*-*MYB113*-*Like* is conserved in grape berry development and anthocyanin accumulation in response to UV-B light due to *VviMYBA6* and *VviMYBA7* being orthologs of *AtPAP1*/*PAP2* and *AtMYB113* (Sunitha et al., 2019). A potential role of *MYB82* in the regulation of anthocyanin was predicted in *Brassica rapa* by (Zhou et al., 2020), whereas a mismatch in the “seed region” of miR828 binding site in At*MYB82* suggested that this gene was of questionable significance as relates to miR828 activities (Rajagopalan et al., 2006) and had yet to be validated in Arabidopsis. Our RNA-seq data showed an upregulation of *MYB82* in response to sucrose (**Table 1**) and publicly available degradome analysis validates *MYB82* is a bona fide sliced target of miR828 (**Figure 6C**). miR858 is a positive regulator of anthocyanin biosynthesis in Arabidopsis and was claimed to exert its regulation through post-translational repression of the negative regulator *MYBL2* in Arabidopsis (Wang et al., 2016) and phasiRNA transitive post-transcriptional silencing of *VvMYB114* in grape (Tirumalai et al., 2019). Our data (**Table 1**) show that miR858 is upregulated in response to sucrose treatment, whereas the anthocyanin repressor *MYBL2* target is anti-concordantly downregulated in response to sucrose. In addition, we show that miR858 can also regulate the expression of *MYBL2* by AGO-mediated slicing (**Figure 6D**). Anthocyanin biosynthesis can be seen as a direct consequence of the excess carbon source in the cells shunted toward secondary metabolite biosynthesis, with upregulation of miR828 and miR858 expression as principal molecular mechanisms because almost all of the activators of the anthocyanin regulatory pathway were upregulated, and repressors were downregulated in response to sucrose treatment (**Figure 5**).

Sucrose signaling and copper homeostasis are two distinct but closely related processes in plants. When exogenous sucrose is applied to the growth medium, various enzymes metabolize the sucrose and many of these biosynthetic enzymes have copper co-factor. Consistent with what was observed by (Ren and Tang, 2012), we also show upregulation of copper-responsive miRNAs miR398 and miR408 and anti-concordant downregulation of their respective cognate targets *CCS1*, *CSD2*, *BCB*, *UCC2*, and *PAA2* (**Table 1**). The downregulation of these copper-binding proteins would result in increased abundance of free copper, which could then be supplied to other metabolic enzymes induced by sucrose. Thus, sucrose plays an important role in copper homeostasis in plants by allocating copper to match growth and metabolic needs under different environmental conditions. Some of the cognate targets (such as Rubredoxin-like superfamily protein and *LAC13*) of miR398 and miR408, respectively, were seen to be upregulated, which could be a homeostatic response to fine-tune the copper availability in the cell. miR408 has been proposed to control light-induced anthocyanin biosynthesis via crosstalk between copper homeostasis and ROS homeostasis (Hu et al., 2023). We identify a direct link between miR408 and anthocyanin biosynthesis by demonstrating that miR408 has a complementary non-canonical binding site in the *F3′H* coding region with an Allen score of 5 and can negatively affect *F3′H* expression through AGO-mediated slicing (**Figure 6A**). *F3′H* is an early anthocyanin biosynthesis gene coordinately expressed with *chalcone synthase* and *chalcone isomerases* (**Figure 4A**, **Supplementary Dataset S6**). Expression of miR408 and *F3′H* were concordantly upregulated in response to sucrose treatment, as is the case for all the genetic activators of anthocyanin biosynthesis pathway and their respective miRNA effectors (**Table 1**). In addition to the previously reported miR828-TAS4:MYBs and miR858:MYBs modules, the newly identified miR408:F3*′*H module adds an extra layer of regulation of carbon flux toward anthocyanin synthesis and accumulation in response to high sucrose.

The *OR* gene is a key effector of chromoplast development, carotenoid biosynthesis, and potentially retrograde signaling based on dominant allele pleiotropic phenotypes characterized in cauliflower/*B. oleracea* (Lu et al., 2006), sweet potato (Park et al., 2015) rice (Endo et al., 2019; Yu et al., 2021), cucumber, and melon (Tzuri et al., 2015; Kishor et al., 2021). *OR* has also been shown to play significant roles in abiotic stress tolerance and shoot development (Shan et al., 2022). Overexpression of *AtOR* represses flowering through the *CO*-*FT*-*SOC1*-mediated photoperiodic flowering pathway in *Arabidopsis* (Wang et al., 2022). In addition to its role in carotenoid accumulation, overexpression of *AtOR* in tomato resulted in alteration of horticultural traits like increased plastid size, early flowering, early fruit ripening, and increased fruit set and seed production (Yazdani et al., 2019). Our analysis revealed a novel non-canonical miR398*: *AtOR* slicing module with an Allen score of 8 (**Figure 6B**). We found that miR398* expression was upregulated in sucrose-treated sRNA library samples and that *AtOR* expression was anti-concordantly downregulated in response to sucrose treatment (**Table 1**), providing additional evidence in support of miR398* function. Because miR398 is a stress-responsive miRNA, upregulation of miR398* in response to sucrose may have caused *AtOR* downregulation. Sucrose treatment can potentially upregulate the carotenoid accumulation, but the molecular mechanism by which sucrose regulates carotenoid metabolism at transcriptional level is still unknown (Durán-Soria et al., 2020). In addition, the exact effect of sucrose on carotenoid biosynthesis, transport, and metabolism is unclear as our results show a significant enrichment of downregulated genes in carotenoid biosynthesis pathway in response to sucrose treatment. Our tantalizing observation suggests a novel miR398*-mediated abiotic stress response and primary carbon biosynthetic mechanisms in Arabidopsis through carotenoid biosynthesis and homeostasis, but the observation needs further independent verification. A broader functional significance in plants for this specific non-canonical ath-miR398*: *OR* interaction module is questionable because we found no evidence for slicing activity or compensatory *OR* target site mutations that would preserve the base pairing, predicted from miR398* divergences and interrogated in publicly available degradome datasets in rice and closely related canola B. *napus* (data not shown). Detailed analysis of deep sRNA (Lunardon et al., 2020; Chen et al., 2021) and degradome datasets developed from different plant tissues and species can critically test novel non-canonical miRNA targets and their evolutionary trajectories correlated to hypothesized co-evolution/diversification of effector *MIRNA* family members.

In addition, various miRNAs like miR156, 160, 167, 172, 319, 395, 397, 398, 399, 408, and 827 have been reported to be differentially expressed in response to carbon starvation as well as other nutrient-deficient stress conditions as catalogued in **Table 1**. Previous genetic and genomic analysis of sugar and amino acid transporters has revealed links between sucrose as a global regulator with pleiotropic effects on nitrogen and P_i_ homeostasis (Lei et al., 2011; Dasgupta et al., 2014; Yadav et al., 2015), consistent with our and others’ (Hsieh et al., 2009) interpretations of miRNAs as nodes in carbon and nutrient crosstalk networks. The interplay between sucrose signaling and miRNA-mediated post-transcriptional gene silencing pathway is reported to regulate growth and development of plants. Several reports show that sugar promotes the vegetative phase change (juvenile to adult transition) by repressing miR156 (Yang et al., 2013a; Meng et al., 2021). miR156a/c:SPL modules play pivotal roles in this process, and we also observed reductions in the accumulation of miR156. In contrast to our working hypothesis, we found a concordant downregulation of miR156 cognate targets (*SPL3* and *SPL10*) but no change in *SPL7* and *SPL9* expression. It is worth noting that the miR156:*SPL9* module and the *PAP1*-*CYTOSOLIC INVERTASE1*/*2* modules interact to promote the juvenile-to-adult transition and that *SPL9* can bind to the promoter of *PAP1* and directly trigger its expression. The observed concordant downregulation of SPLs and miR156 could be explained as a result of above-mentioned independent interaction of SPLs and *PAP1*, as *PAP1* induction upon sugar treatment could negatively affect *SPL* gene expression (Cui et al., 2014; Wang et al., 2020; Li X. et al., 2021; Meng et al., 2021). The observed genotype-by-sucrose interaction effect on miR156 accumulation in *pap1-D*–treated seedlings can, thus, be explained by the interplay between *PAP1* and *SPL*s. Furthermore, we found apparent and significant genotype-by-sucrose interactions (compare **Figure 2A** column clustering with **Supplementary Dataset S3**, column P) on significantly DE miR156bd, miR158a, miR162a, miR169b, miR171b, miR391, miR397a, miR399f, and miR846 accumulations in *pap1-D* treated seedlings, indicating that *PAP1* might also act an effector on these miRNAs’ known targets.

Last, our analysis found that under high sucrose stress several mRNA targets were mis-regulated in a concordant manner with their miRNA effector DE (**Table 1**), suggesting molecular mechanisms may be involved other than observed miRNA abundances as proxy for inferred AGO slicing activities. Although more common in animal than its counterpart plant, a possibility could be that the targets undergo miRNA-mediated translational inhibition rather than cleavage and the mRNA levels would be maintained constant under these specific stress conditions. Some miRNAs can exert coexistence of cleavage and translational repression on the same target gene as in the case of miR398 and its target *CSD1*, *CSD2*, and *CCS1* (Dugas and Bartel, 2008; Beauclair et al., 2010). However, more research is needed to decipher how each mode is decided under different growth and stress condition (Yang et al., 2021).

## Data availability statement

The datasets presented in this study can be found in online repositories. The names of the repository/repositories and accession number(s) can be found below: https://www.ncbi.nlm.nih.gov/, PRJNA995345.

## Author contributions

MA: Conceptualization, Data curation, Formal Analysis, Investigation, Writing – original draft. PD: Conceptualization, Data curation, Formal Analysis, Investigation, Methodology, Writing – original draft, Writing – review & editing. NE: Investigation, Writing – review & editing. CR: Conceptualization, Data curation, Formal Analysis, Investigation, Methodology, Writing – original draft, Writing – review & editing.
