# Supplementary material for: Role of miRNAs in sucrose stress response, reactive oxygen species, and anthocyanin biosynthesis in Arabidopsis thaliana
Source: Front Plant Sci. 2023 Nov 3;14:1278320. doi: 10.3389/fpls.2023.1278320 (PMC10656695; doi:10.3389/fpls.2023.1278320)
Supplement: Supplementary file 1 [file DataSheet_1.docx]

**Supplemental File: Methods and Bioinformatics, Table, and Figures**

**Supplemental Table S1:** Oligos used in small RNA Northern blotting

| **Oligo Name** | **No. of bases** | **Sequence** |
| --- | --- | --- |
| miR398-anti | 21 | CAGGGGTGACCTGAGAACACA |
| miR408-anti | 21 | GCCAGGGAAGAGGCAGTGCAT |
| TAS4-3pD4-_anti | 21 | TGCCTCGACCTCGATCCTTCA |
| miR828-LNA-anti (+ indicates locked nucleic acid chemistry) | - | T+GG+AATACTCATTTGAGC+AA |
| Anti-5S rRNA | 23 | AGGACTTCCCAGGAGGTCACCCC |

**Supplemental Figures**

**
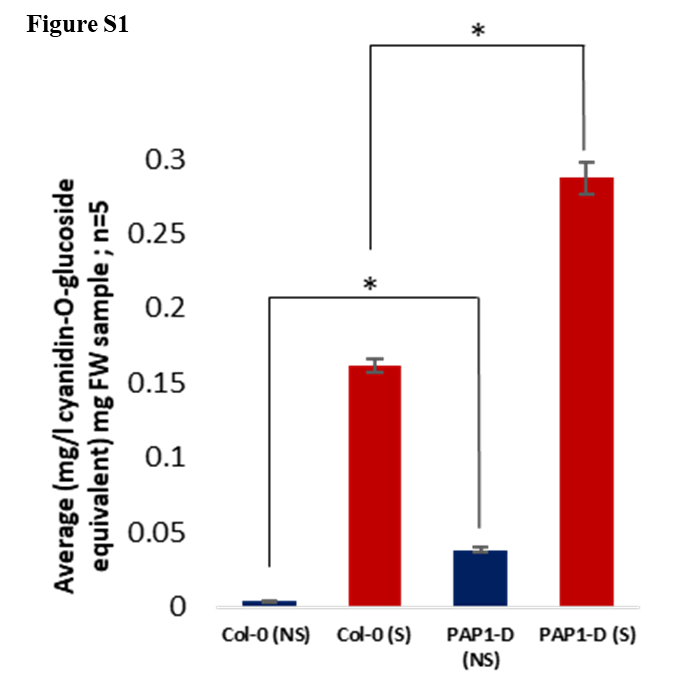
**

**Supplemental Figure S1.** Anthocyanin quantitation of sucrose treated and untreated Col-0 and *pap1-D* seedlings. Asterisk (*) indicates PAP1-D significantly different from Col-0 genotype in both untreated (NS) and high sucrose-(S) treated samples, *p* < 0.05 (Student’s t-test, equal variance assumed). Error bars are s.e.m.

**
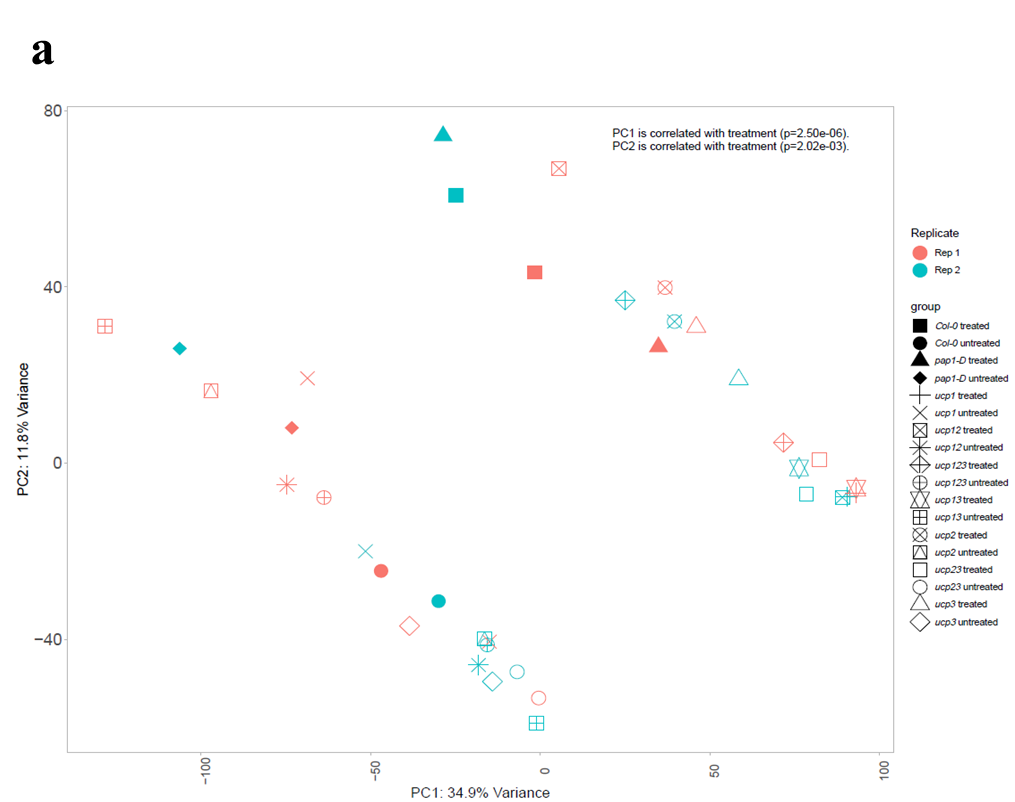
**

**
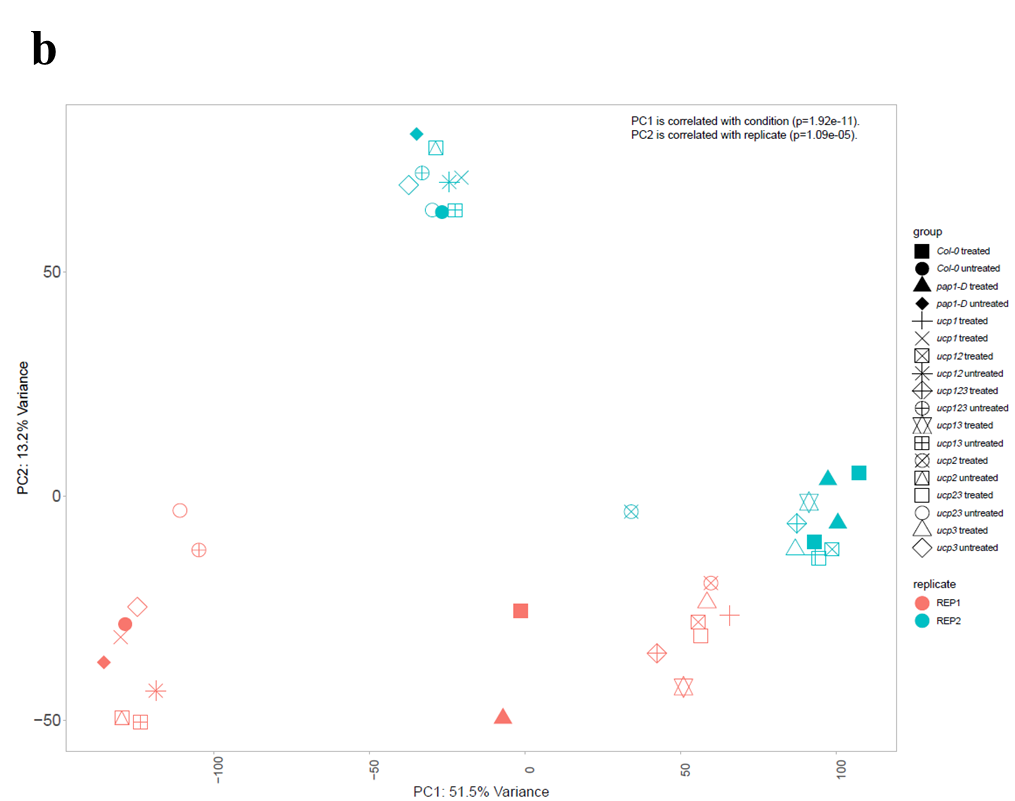
**

**Supplemental Figure S2** (above)**:** Principal Component Analysis (PCA) of small RNA-seq and RNA-seq datasets. **a)** Principal Component Analysis (PCA) of small RNA-seq datasets from Col-0, *pap1-D*, *ucp1*, *ucp2*, *ucp3*, *ucp12*, *ucp13*, *ucp23*, and *ucp123* sucrose treated and untreated samples. PC1 and PC2 dimensions correlated with the sucrose treatment and captures ~35% and ~12% of the variation in the study samples at p-values of 2.5e-6 and 2.02e-3, respectively. small RNA-seq PCA plot. **b)** Principal Component Analysis (PCA) of RNA-seq datasets from Col-0, *pap1-D*, *ucp1*, *ucp2*, *ucp3*, *ucp12*, *ucp13*, *ucp23*, and *ucp123* sucrose treated and untreated samples. PC1 and PC2 dimensions correlated with the sucrose treatment and replicate respectively and captures ~52% and ~13% of the variation in the study samples at p-values of 1.92e-11 and 1.09e-6, respectively.

**
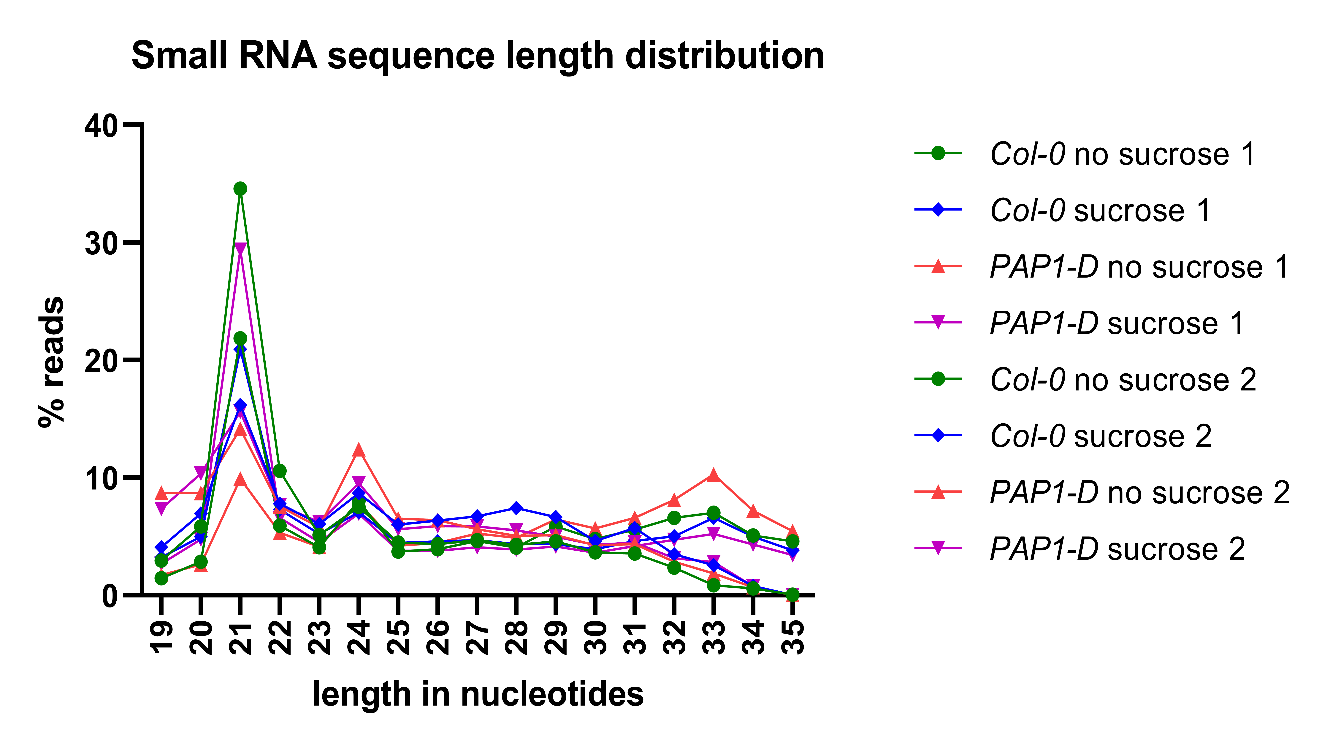
**

**Supplemental Figure S3:** Length distribution of small RNAs. Data point/lines are from biological duplicates made at six-day seedling stage sucrose-treated and untreated samples in Col-0 and *pap1-D* genetic background. Y-axis represents the percentage of reads and x-axis represents the length in nucleotides.

**
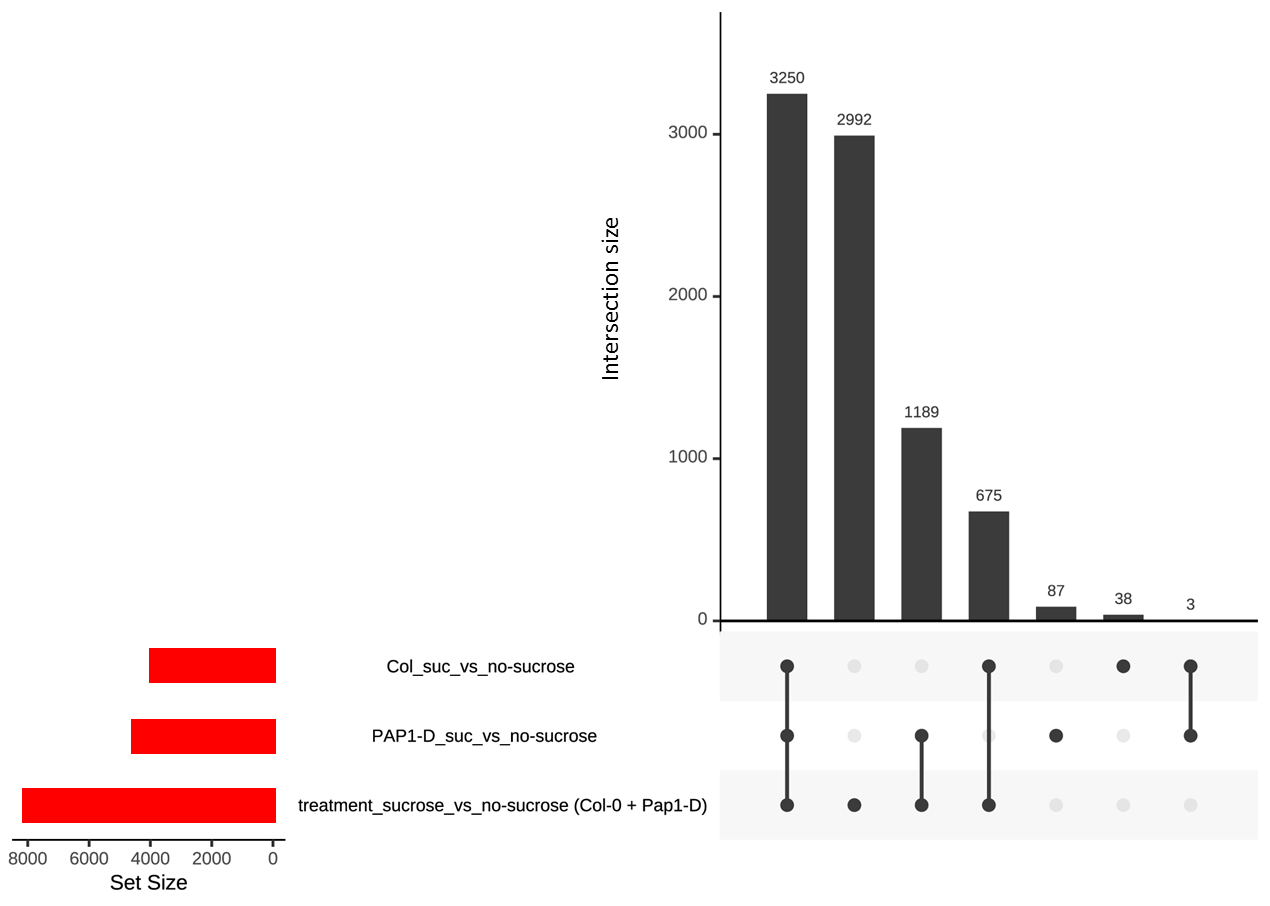
**

**Supplemental Figure S4:** UpSet plot displaying the number of significantly differentially expressed transcripts (p-adjusted < 0.05) shared among overall sucrose effect (Col-0 + *pap1-D*), sucrose effect in Col-0 and *pap1-D* backgrounds.

**Supplemental Materials and Methods**

**Anthocyanin quantification**

Anthocyanin pigment (cyanidin-3-glucoside equivalents, mg/L) = A x MW x DF x 10^3^ /ε x 1 x sample weight in mg, where A = (A520nm – A700nm) pH 1.0 – (A520nm – A700nm) pH 4.5, MW (molecular weight) = 449.2 g/mol for cyanidin-3-glucoside (cyd-3-glu), DF = dilution factor, 1 = pathlength in cm, ε = 26,900 molar extinction coefficient in L x mol ^–1^ x cm^–1^, for cyd-3-glu and 10^3^ = factor for conversion from g to mg.

**RNA and small RNA library preparation and sequencing**

One μg of total RNA was taken forward per sample to prepare RNA-seq libraries by Illumina TruSeq Stranded Total RNA with RiboZero Plant RNA-seq kit (catalogue #20020610) with time-dependent hydrolysis step to yield an average RNA fragment size of 150 nt according to the manufacturer’s protocol. Unique dual indexes (Illumina IDT UD RNA, catalogue # 20020591) were used for RNAseq samples and finished libraries were run on 6% native PAGE gel and bands between ~ 260 to 310 bp were excised and subjected to gel breaking (Thermo Fisher Scientific 1st Engineering Gel Breaker Tubes). The gel clean-up applied to only the first biological replicate of RNA seq library prep; and the second round of library prep were directly run in the Bioanalyzer for quality control check. 50 ng of extracted sRNA was used to prepare libraries using Illumina TruSeq Small RNA library preparation kits with index sets A-C (catalogue #RS-200-0012, -0024, -0036) according to manufacturer’s protocols, and bands between ~ 145 to 160 bp were excised from native PAGE gel and subjected to gel breaking. The completed sRNA and RNAseq libraries were quantified and qualified on an Agilent 2100 Bioanalyzer using High-Sensitivity DNA kit (catalogue #5067-4626).

Two equimolar pools of 18 RNA-seq indexed biological duplicate libraries were prepared and subjected to adapter blocking reagent (Illumina #20024144) and sequentially sequenced to yield 2 x 50 bp, and 2 x 150 bp, paired reads on Illumina NovaSeq 6000 S2 and S4 platforms by the UC Riverside Institute for Integrated Genome Biology Core facility, respectively for biological replicates. The RNA-seq libraries were sequenced twice sequentially as technical replicates, with output files combined after verification by DESeq2 there was no significant difference between technical replicate counts. Similarly, two equimolar 18 sRNA-seq indexed libraries (biological duplicates, except an additional *ucp1* experiment 1 no sucrose control library was repeated due to low yields) were prepared and sent for sequencing to yield 1 x 75 bp single end, and 2 x 37 bp paired end reads for biological replicates, respectively on Illumina NextSeq500 platform by the UC Riverside Institute for Integrated Genome Biology Core facility.

**Sequence Data and Bioinformatics analyses**

RNA-seq and sRNA-seq libraries were quality assessed using FastQC v0.11.5 (https://www.bioinformatics.babraham.ac.uk/projects/fastqc/). The sRNA libraries were subjected to 3’ sRNA adapter “TGGAATTCTCGGGTGCCAAGG” trimming with fastx_clipper tool of the FASTX toolkit (http://hannonlab.cshl.edu/fastx_toolkit/index.html) and reads with length greater than 18 bp were retained. In case of any adapter contamination in the RNA-Seq data, adapter clipping was performed using Trimmomatic (Bolger et al., 2014) with default parameters i.e. ILLUMINACLIP:TruSeq3-PE.fa:2:30:10:2:True LEADING:3 TRAILING:3 MINLEN:36.

ShortStack output provides “MajorRNA” species and “complexity” metric parameters for each identified cluster. ShortStack clusters were further annotated to known miRNA generating loci using BLASTn algorithm (Altschul et al., 1990) where the “MajorRNA” was blasted against a known miRNA database for *Arabidopsis thaliana* downloaded from miRbase which was further verified using the genomic coordinates. Generally, MajorRNA species corresponds to mature miRNA species (or star, when it is claimed to be functional), although in some cases, a few nucleotide overhangs were identified that may have been caused by DICER sloppiness generating isoMIRs (see **Supplementary Dataset S3**). A complexity parameter is calculated by dividing the number of distinct sRNA alignments by total abundance of sRNA alignments for a sRNA cluster. Since the average complexity of the sRNA clusters annotated as known miRNA-generating clusters was very low (~0.08, with several loci having zero complexity, see **Supplementary Dataset S3**), differential abundances of the sRNA clusters are appropriate proxy for quantifying the mature miRNA species Counts for downstream differential expression analysis. Complexity averaged 0.086 for PHASI loci across all claimed loci. For *TAS4*, the functional 3′-D4(-) siRNA was the major species and Complexity was 0.017. Taken together, these data support that statistical inference of miRNA and siRNA dynamics using clusters as proxy for bona fide effectors is reasonable and useful, when quality assured as documented in **Supplemental Dataset S3**.

The raw counts for gene loci generated by Kallisto in each RNA-seq library and raw counts for the top 25,000 sRNA-generating loci based on abundance (**Supplemental Dataset S3**) accounting for all identified/annotated *MIRNA* loci, sRNA-generating loci with a phase score of 30 or above, and remaining top sRNA-generating clusters sorted based on descending mean read abundance (cutoff ≥ 0.177 reads per million) were utilized as an input for respective differential expression analysis in DESeq2 R package (release 3.14) (Love et al., 2014).

**Supplemental Reference**

**Altschul SF, Gish W, Miller W, Myers EW, Lipman DJ** (1990) Basic local alignment search tool. Journal of Molecular Biology **215**: 403-410

**Bioinformatic scripts**

**Fastx-clipper**

./fastx_toolkit-0.0.14/bin/bin/fastx_clipper -a TGGAATTCTCGGGTGCCAAGG -v -c -l 19 -i /path_to_input_file -o path_to_output_file

**Bowtie**

/bowtie-1.1.2/bowtie -a -v 2 -l 19 --best --strata path_to_index -q path_to_query_input_fastq_file --al path_to_aligned_output_fastq_file --un /path_to_unaligned_output_fastq_file

**Trimmomatic**

module load java

java -jar ./Trimmomatic-0.38/trimmomatic-0.38.jar PE path_to_input_R1_fastq_file path_to_input_R2_fastq_file path_to_output_R1_paired_fastq_file path_to_output_R1_unpaired_fastq_file path_to_output_R2_paired_fastq_file path_to_output_R2_unpaired_fastq_file ILLUMINACLIP./Trimmomatic-0.38/adapters/TruSeq3-PE-2.fa:2:30:10:8:True

**Kallisto**

./kallisto_linux-v0.43.1/kallisto quant -i path_to_index -o path_to_output_directory -b 100 path_to_cleaned_input_R1_file path_to_cleaned_input_R2_file

**ShortStack**

module load perl/5.16.3

./ShortStack-3.8.5/ShortStack –readfile path_to_all_input_fastq_files --genomefile path_to_genome_index --bowtie_cores 24 --foldsize 1000 --sort_mem 160G --outdir path_to_output_directory

**CleaveLand**

module load perl/5.16.3

cd ./CleaveLand4-master/

perl CleaveLand4.pl -e path_to_input_degradome_fasta_file -n path_to_cDNA_index -u path_to_mature_miRNA_fasta_file -r 0.60 -o path_to_cleaveland_output_directory -t > path_to_cleaveland_tabular_output

**PhaseTank**

module load perl/5.16.3

cd ./PhaseTank_Tutorial/

perl PhaseTank_v1.0.pl --genome path_to_genome_index --lib path_to_input_collapsed_sRNA_input_fasta_file --miR /path_to_mature_miRNA_fasta_file --degradome path_to_input_degradome_fasta_file --target path_to_cDNA_index --rsrp -3.293 --phasiRNA_target --trigger_miRNA --dir path_to_phasetank_output_directory

**DESeq2 R package**

library("DESeq2")

FP <- read.delim(file="path_to_input_file", header=TRUE, row.names = 1)

countData = data.frame(FP)

columnData <- read.delim(file="path_to_metadata_file", row.names=1)

all(rownames(columnData) %in% colnames(countData))

countData <- countData[, rownames(columnData)]

all(rownames(columnData) == colnames(countData))

ddFP<- DESeqDataSetFromMatrix(countData = round(countData), colData = columnData, design =~genotype+batch+condition)

ddFP

ddFP$genotype <- factor(ddFP$genotype, levels = c(..))

ddFP$genotype <- relevel(ddFP$genotype, ref = "reference_genotype_library")

ddFP$condition <- relevel(ddFP$condition, ref = "untreated")

ddFP <- DESeq(ddFP)

resultsNames(ddFP)

results(ddFP)

res1 <- results(ddFP, name = "name_of_desired_comparison")

write.csv(as.data.frame(res1), file="name_of_the_file.csv")
